# Sequencing of 231 forensic genetic markers using the MiSeq FGx™ forensic genomics system – an evaluation of the assay and software

**DOI:** 10.1080/20961790.2018.1446672

**Published:** 2018-04-09

**Authors:** Christian Hussing, Christina Huber, Rajmonda Bytyci, Helle S. Mogensen, Niels Morling, Claus Børsting

**Affiliations:** Section of Forensic Genetics, Department of Forensic Medicine, Faculty of Health and Medical Sciences, University of Copenhagen, Copenhagen, Denmark

**Keywords:** Forensic science, forensic genetics, next generation sequencing, short tandem repeats, single nucleotide polymorphisms, ForenSeq™ DNA Signature Prep Kit, MiSeq FGx™ Forensic Genomics System

## Abstract

The MiSeq FGx™ Forensic Genomics System types 231 genetic markers in one multiplex polymerase chain reaction (PCR) assay. The markers include core forensic short tandem repeats (STRs) as well as identity, ancestry and phenotype informative short nucleotide polymorphisms (SNPs). In this work, the MiSeq FGx™ Forensic Genomics System was evaluated by analysing reproducibility, sensitivity, mixture identification and forensic phenotyping capabilities of the assay. Furthermore, the genotype calling of the ForenSeq™ Universal Analysis Software was verified by analysing fastq.gz files from the MiSeq FGx™ platform using the softwares STRinNGS and GATK. Overall, the performance of the MiSeq FGx™ Forensic Genomics System was high. However, locus and allele drop-outs were relatively frequent at six loci (two STRs and four human identification SNPs) due to low read depth or skewed heterozygote balances, and the stutter ratios were larger than those observed with conventional STR genotyping methods. The risk of locus and allele drop-outs increased dramatically when the amount of DNA in the first PCR was lower than 250 pg. Two-person 50:1 mixtures were identified as mixtures, whereas 100:1 and 1 000:1 mixtures were not. Y-chromosomal short tandem repeats (Y-STRs) alleles were detected in the 100:1 and 1 000:1 female/male mixtures. The ForenSeq™ Universal Analysis Software provided the data analyst with useful alerts that simplified the analysis of the large number of markers. Many of the alerts were due to user-defined, locus-specific criteria. The results shown here indicated that the default settings should be altered for some loci. Also, recommended changes to the assay and software are discussed.

## Introduction

For more than 15 years, forensic DNA profiles have been generated by polymerase chain reaction (PCR) amplification of the core forensic short tandem repeats (STRs) and by detection of the PCR products with capillary electrophoresis (CE) [1]. PCR–CE typing proved to be highly efficient and relatively cheap. However, it has limited multiplexing capability, and the STR analyses cannot easily be combined with typing of other types of forensic loci, e.g. short nucleotide polymorphisms (SNPs), indels, mtDNA or RNA, in the same assay. With the introduction of PCR-based next generation sequencing (NGS) methods in forensic genetics [2–13], a new method for the analysis of PCR products was made available that may surmount many of the limitations of PCR–CE. The multiplexing capacity of the current PCR–NGS assays far exceeds the number of classical forensic genetic markers, and different types of loci (SNPs, indels, STRs or any combination of SNP–STR–indel haplotypes) may be typed in the same multiplex assay. Furthermore, the amplification products need not to be separated by colour and/or size as in PCR–CE assays, because the loci can be identified by their DNA sequences. Thus, the PCR amplicons can be designed to be as short as possible, which may improve the probability of typing degraded DNA. Finally, the complete sequence variations of the loci are revealed, which increases the statistical weight of the DNA evidence and may improve the resolution of mixtures [4].

The MiSeq FGx™ Forensic Genomics System includes the ForenSeq™ DNA Signature Prep Kit, the MiSeq FGx™ sequencing instrument and the ForenSeq™ Universal Analysis Software (UAS) [11]. The ForenSeq™ DNA Signature Prep Kit is one of the first commercial kits for simultaneous typing of forensically relevant STRs and SNPs. The loci may be amplified with one of two primer mixes: Primer Mix A amplifies loci for human identification (HID), while Primer Mix B amplifies the same HID loci along with ancestry informative markers (AIMs) as well as markers associated with eye and hair colour. The MiSeq FGx™ Forensic Genomics System types 27 autosomal STRs, 7 X-STRs, 24 Y-chromosomal short tandem repeats (Y-STRs), amelogenin and 94 HID SNPs that were specifically selected for HID purposes by the forensic community [14–21]. The STRs include the 13 combined DNA index system (CODIS) core loci [22], the 12 European Set of Standard (ESS) markers [1] and 6 of the 7 Y chromsome STR haplotype reference database (YHRD) core loci in the minimal Y-STR haplotype [23], which ensure comparability with the DNA profiles in already existing DNA databases. The kit also amplifies 56 AIM SNPs [24] as well as 24 eye and hair colour predictive SNPs [25]. In total, 231 markers relevant for forensic genetics are sequenced. The amplicon lengths of the STRs and SNPs are in the ranges 61–467 and 63–231 bp, respectively.

Previous studies have already assessed the performance of the MiSeq FGx™ Forensic Genomics System [6,11,26–30]. In this study, we evaluated the system by assessing the read depth of the markers, allele balances and stutter and noise ratios using the fastq files from the MiSeq FGx™ platform. We compared these analyses with the results generated by the ForenSeq™ UAS and evaluated the reports generated by the ForenSeq™ UAS. Furthermore, we analysed the sensitivity of the workflow and the abilities of the ForenSeq™ UAS to identify DNA mixtures, and to predict biogeographical ancestry as well as eye and hair colour.

## Materials and methods

### Samples and DNA extraction

Material from buccal swabs was collected on FTA cards from 12 Danes and 1 Argentinian individual. The DNA was extracted from two 3 mm punches using the Trace Tip Dance (TD) procedure of the EZ1 DNA Investigator Kit (Qiagen, Hilden, Germany) and the EZ1 Advanced XL Instrument (Qiagen). The extracted DNA was eluted in 50 µL water.

Blood samples from 10 Italians, 2 Ethiopians and 7 individuals of Eastern European, Moroccan, Iraqi, Indian, Korean, Chinese and Brazilian origin, respectively, were used in this study. The DNA was extracted from 200 µL blood using the spin protocol of the QIAamp® DNA Mini Kit (Qiagen). The DNA extractions were quantified using the Qubit 3.0 instrument (Thermo Fisher Scientific, Waltham, MA, USA).

### STR typing with CE

DNA extracts from 30 individuals were typed for 15 STR loci (*D1S1656*, *D2S1338*, *D2S441*, *D3S1358*, *D8S1179*, *D10S1248*, *D12S391*, *D16S539*, *D18S51*, *D19S433***,***D21S11***,***D22S1045*, *FGA*, *TH01* and *vWa*) and the amelogenin locus using the AmpFℓSTR® NGMSElect Express PCR Amplification Kit (Thermo Fisher Scientific) and the AB3500*xl* instrument (Thermo Fisher Scientific). Laboratory procedures and data interpretation using GeneMapper ID-X v.1.4 were done according to the accredited workflow for paternity case samples at the Department of Forensic Medicine, University of Copenhagen [31].

### Library building, MiSeq FGx™ sequencing and data analysis

Libraries were built using the ForenSeq™ DNA Signature Prep Kit (Illumina®) following the manufacturer's protocol. Four library pools were made. Pool 1 contained libraries from 10 Danes, 10 Italians and 10 individuals from various countries (Table 1). Pool 2 contained libraries from the same individuals as Pool 1. The libraries in Pools 1 and 2 were constructed independently of each other. Pool 3 contained libraries for a sensitivity study. Dilution series of DNA from four individuals (four Danes including two individuals that were not typed in Pools 1 and 2) were made. The amount of DNA in the first PCR was 1 000, 500, 250, 125, 62.5, 31.3, 15.6 and 7.8 pg, respectively. Each dilution in Pool 3 was amplified and sequenced three times. Pool 4 contained libraries for a mixture study. Fifteen mixtures (1:1 000, 1:100, 1:50, 1:25, 1:12, 1:6, 1:3, 1:1, 3:1, 6:1, 12:1, 25:1, 50:1, 100:1 and 1 000:1) were constructed from samples from a male and a female individual, respectively. Each mixture sample was amplified and sequenced two times. Except for Pool 3, 1 ng DNA was used in the first PCR. Primer Mix B was used for amplification of the samples in Pools 1, 2 and 4, whereas Primer Mix A was used for amplification of the samples in Pool 3. Pools 1, 2 and 4 included 32 libraries, of which 2 were the positive control 2800M and a negative PCR control, while Pool 3 included 96 libraries. The library pools were sequenced on the MiSeq FGx™ instrument (Illumina®) following the protocol of the manufacturer. The run quality metrics of the sequencing (cluster density, clusters passing filter, phasing and pre-phasing) were all within the recommended range (data not shown). In Pools 1, 2 and 4, the read depth ranged from 136 381 to 754 858 reads per sample with a median read depth of 339 855; 432 871 and 308 877 reads, respectively. The cluster densities were 1 197, 1 376 and 1 453 k/mm^2^, and 92.8%, 91.3% and 88.8% of the clusters passed the MiSeqFGx™'s “cluster passing filter” in Pools 1, 2 and 4, respectively. In Pool 3, the read depth ranged from 983 to 102 329 reads per sample with a median of 18 398 reads. The cluster density was 665 k/mm^2^, and 96.8% of the clusters passed the “cluster passing filter”. Overall, the sample read depth decreased as the amount of input DNA decreased. All sequencing runs were analysed with the ForenSeq™ UAS [32]. The “Project Detail Report” offered by the ForenSeq™ UAS was used for further analysis.

**Table 1.** Geographical origin of the individuals sequenced in Pools 1 and 2 on the MiSeq FGx™ instrument.

Continental origin	National origin	Number of samples
European	Danish	10
	Italian	10
	Eastern European	1
African	Ethiopian	2
North African and Middle Eastern	Moroccan	1
	Iraqi	1
South Asian	Indian	1
East Asian	Korean	1
	Chinese	1
South American	Brazilian	1
	Argentinian	1

### STRinNGS STR analysis

Fastq.gz files from the MiSeq FGx™ sequencing were mapped to hg19 (GRCh37) using an in-house script. The resulting BAM files were analysed with the STRinNGS v.1.0 software [5]. In short, STRinNGS sorted the reads from the BAM files according to the barcodes assigned to each individual. For each barcode, the reads for each STR system were subsequently sorted via their specified flanking sequences, and each sequence read was parsed according to the specified nomenclature. Configuration files stated the length of the flanking regions as well as the repeat structure according to information in the STRbase (http://www.cstl.nist.gov/strbase/) and the literature [14–19,33–35] (Supplementary File 1). The nomenclature described by Gelardi et al. [36] was applied. Forward strand repeat structure was used in accordance with the recommendations of the International Society for Forensic Genetics [35]. The main output file contained a description of all sequences with read counts above 1% of the total number of reads per locus.

STRinNGS output was used to determine the read depth, heterozygous balance (Hb) and fractions of stutter reads based on duplicate sequencing of 30 single contributor samples. The Hb for STRs was calculated as the read count for the longest allele divided by the read count for the shortest allele.

### GATK SNP analysis

Reads obtained from the fastq.gz files from the MiSeqFGx™ instrument were trimmed to a *Q*-score of 30 using AdapterRemoval v.2.1.3 [37]. Reads were mapped to hg19 using BWA-MEM (http://bio-bwa.sourceforge.net/) and aligned reads were extracted using SAMtools [38]. Genotypes were called with GATK 3.6 (https://software.broadinstitute.org/gatk/) using the default genotyping procedure [39]. Alleles with read depth below 20 reads were discarded. Locus read depth, Hb and noise levels were identified and plotted using in-house Python scripts. Hb was calculated as the number of reads of one nucleotide divided by the number of reads of the other nucleotide in the order A, C, G and T. Noise was calculated as the number of reads that was different from the true genotype divided by the total number of reads.

### Ancestry and phenotype predictions

The ForenSeq™ UAS ancestry predictions were compared to self-reported ancestry. The ForenSeq™ UAS eye colour predictions were compared to the Pixel Indix of the Eye (PIE)-scores obtained with digital photographs and the DIAT software [40]. The ForenSeq™ UAS hair colour predictions were compared to self-reported hair colours, which were available for all study subjects except for the individuals of Eastern European, Iraqi, Indian and Brazilian origin.

## Results

### STR typing using the MiSeq FGx™ forensic genomics system

A total of 30 samples were sequenced in duplicate with the ForenSeq™ DNA Signature Prep Kit using Primer Mix B (Pools 1 and 2) and analysed with the ForenSeq™ UAS. All 58 and 34 STR markers were typed in all samples from male and female individuals, respectively. However, *DXS10103* was not called in one of the duplicate typings of two samples because of low read depth (≤30 allele read counts).

A total of 1308 STRs (58 STRs typed in 12 male samples and 34 STRs typed in 18 female samples) were sequenced twice, and 1290 genotypes (98.6%) were reproduced. The details of the 18 genotypes that were not reproduced are shown in Supplementary File 2. Besides the two locus drop-outs in *DXS10103*, discordant genotypes were observed in six loci (*D9S1122*, *D17S1301*, *D20S482*, *D21S11*, *DYS392* and *DXS10135*). In 13 incidents, the discordance was caused by allele drop-in. The drop-in alleles had markedly lower read depth than the other allele(s), and the lengths and sequences of the drop-in alleles indicated that the alleles were stutter artefacts. Only in one incident, the 10,13 genotype call in *DYS392*, the drop-in allele had an unusual length. A total of four allele drop-outs, which were all in *DXS10103*, were observed. In all incidents, sequences of the drop-out allele were present. However, the alleles were not called because the numbers of reads were below the interpretation threshold (IT) defined in the ForenSeq™ UAS.

All 30 samples were also typed with PCR–CE using the AmpFℓSTR® NGMSElect Express PCR Amplification Kit. Of the 450 STR genotypes, 448 (99.6%) were concordant with the genotypes obtained using the ForenSeq™ DNA Signature Prep Kit. Two discordances were caused by drop-ins in the PCR–NGS assay (Supplementary File 3).

### STR sequence analysis

**Figure 1.**
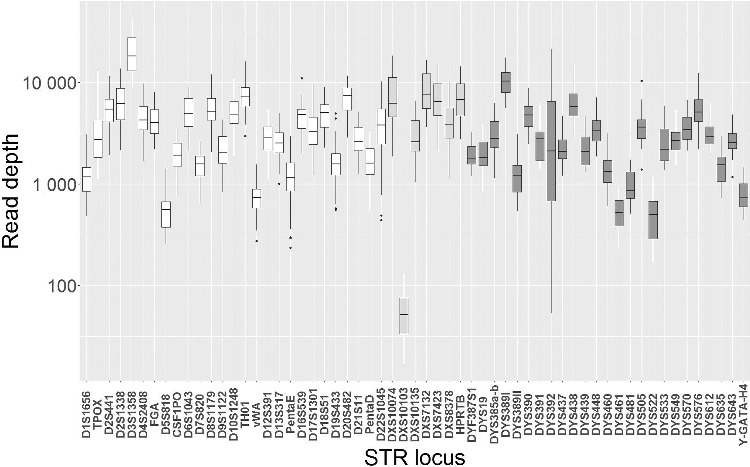
Box-and-whisker plots of the read depth variation of STR loci. Autosomal STRs are marked with white colour, X-chromosomal STRs with pale grey colour, and Y-chromosomal STRs with dark grey colour. The lower and upper limits of the boxes correspond to the 0.25 and the 0.75 quartiles, respectively, and the median is indicated with a bold line. The ends of the whiskers correspond to the most extreme data-point within 1.5 times the interquartile range from the ends of the box. Outliers are indicated by dots.

The locus balance, Hb and fraction of stutter artefacts for each STR locus were analysed using STRinNGS [5] and the fastq files from the duplicate typing of 30 samples (Pools 1 and 2). STRinNGS uses the flanking sequences to sort the reads according to locus. For most loci, the numbers of allowed mismatches in the flanking sequences were set to no more than three. However, more than three mismatches in the flanking sequences were needed to identify reads of *D1S1656*, *D19S433*, *HPRTB* and *DYS522* (Supplementary File 1), indicating that some flanking sequences included a relatively high number of sequencing errors or true variants. The read depth of the Y-chromosome STR *DYS461* is shown in Figure 1. This locus is not included in the list of markers amplified by the ForenSeq™ Signature Prep Kit. However, *DYS461* was amplified on the same fragment as *DYS460* [6], and was readily analysed by STRinNGS (Supplementary File 1).

The median read depth of all STRs was 3 145 reads. The median read depth varied from 52 to 18 164 reads for the various loci (Figure 1). Two loci, *DYS389I* and *D3S1358*, had median read depth above 10 000 reads, whereas *DXS10103* had a median read depth of only 52 reads. The coefficient of variation of the STRs’ read depth varied from 30% to 120%, and was highest for the Y-chromosome STR *DYS392* (Table 2).

**Table 2.** Median and variation of the read depth of the 59 STR loci.

Locus	Median	SD	CV (%)	Locus	Median	SD	CV (%)
*D3S1358*	18 164	8 293	40.7	*TPOX*	2 714	2 438	69.2
*DYS389I*	10 319	3 322	31.3	*DYS549*	2 666	1 081	36.7
*DXS7132*	7 540	3 709	41.8	*DXS10135*	2 610	1 483	47.6
*D20S482*	7 353	2 277	31.8	*D21S11*	2 602	970	34.0
*TH01*	7 332	2 627	34.5	*DYS643*	2 544	884	32.1
*HPRTB*	6 803	3 257	43.9	*D13S317*	2 536	927	35.0
*DXS7423*	6 440	3 360	46.4	*DYS533*	2 189	1 133	44.3
*DXS10074*	6 214	4 460	54.9	*DYS392*	2 119	5 368	120.2
*D2S1338*	6 180	2 749	41.8	*DYS439*	2 091	925	40.2
*DYS438*	57 477	2 931	43.8	*DYS437*	2 081	928	40.0
*D2S441*	5 442	2 109	36.4	*D9S1122*	2 040	848	37.7
*D8S1179*	5 202	2 002	35.7	*CSF1PO*	1 912	661	33.0
*DYS576*	5 097	2 352	41.6	*DYS19*	1 819	839	41.4
*D18S51*	5 043	1 628	32.1	*DYF387S1*	1 770	599	30.1
*D6S1043*	4 939	1 964	36.9	*Penta D*	1 606	674	38.8
*D10S1248*	4 837	1 857	35.2	*D19S433*	1 572	840	48.4
*D16S539*	4 804	1 595	34.2	*D7S820*	1 571	476	30.3
*DYS390*	4 765	1 755	35.1	*DYS635*	1 539	597	38.5
*D4S2408*	4 261	1 912	40.8	*DYS460*	1 325	557	39.2
*FGA*	4 023	1 521	34.5	*DYS389II*	1 218	572	44.4
*DXS8378*	3 852	2 299	51.7	*D1S1656*	1 174	521	41.9
*D22S1045*	3 812	2 418	58.4	*Penta E*	1 158	629	49.9
*DYS505*	3 611	1 836	46.7	*DYS481*	855	359	36.4
*DYS570*	3 411	1 310	34.9	*vWA*	737	309	38.9
*DYS448*	3 339	1 509	40.2	*Y-GATA-H4*	727	307	37.9
*D17S1301*	3 296	1 583	43.1	*D5S818*	560	236	41.9
*DYS612*	2 903	1 241	37.3	*DYS461*	525	213	38.9
*D12S391*	2 871	986	33.9	*DYS522*	498	272	51.9
*DYS385a/b*	2 807	1 358	41.3	*DXS10103*	52	28	49.9
*DYS391*	2 781	1 397	49.0				

SD: standard deviation; CV: coefficient of variation.

**Figure 2.**
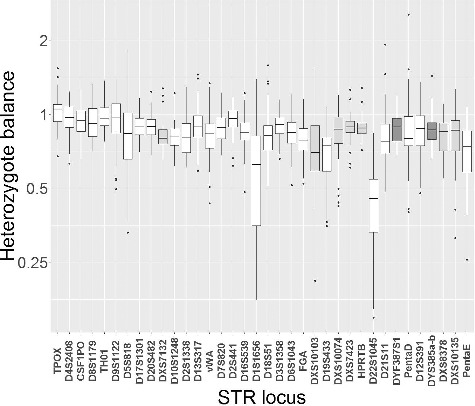
Heterozygote balances of the STRs. The loci are listed according to increasing PCR amplicon size from left to right. Autosomal loci are marked with white colour, X-chromosomal loci with pale grey colour and Y-chromosomal loci with dark grey colour (two amplicons are generated from each of the Y-STRs *DYF387S1* and *DYS385a/b*). The box-and-whisker plot parameters are described in Figure 1.

Hb was calculated in all heterozygous genotypes as the read count of the longest allele divided by the read count of the shortest allele (Figure 2). For most loci, the median Hb was close to or slightly smaller than 1.0, which indicates that the sequencing assay had a tendency to favour the amplification of the shorter allele of a locus. *D22S1045* was the only STR locus with a median Hb < 0.5 (based on 44 heterozygous *D22S1045* genotypes). The largest variation of Hb was observed in *D1S1656* (Hb range: 0.18–1.38). The large Hb variation was caused by microvariant alleles with a [TCA] trinucleotide that interrupted the *D1S1656* [TCTA] repeats. These alleles had lower read counts (approximately one-third) than the alleles without the trinucleotide (data not shown).

**Figure 3.**
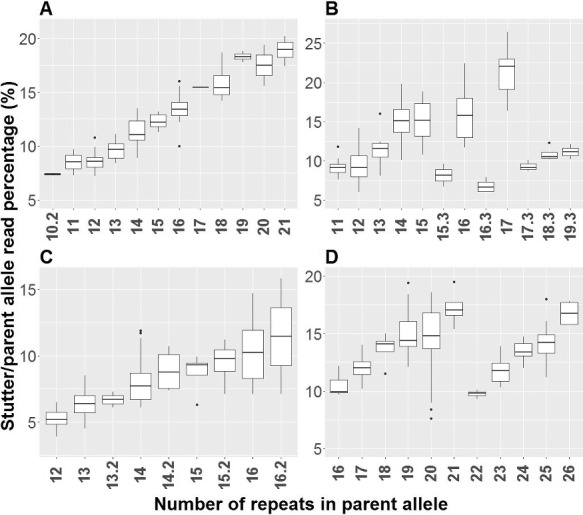
Correlation between the number of STR repeats of the parent alleles and the stutter ratio in per cent at four loci: *D18S51* (A), *D1S1656* (B), *D19S433* (C) and *D2S1338* (D).

The fraction of stutter reads that were one repeat unit shorter than the parent allele (*n* − 1 stutter) was calculated for each allele (Table 3, Figure 3, Supplementary File 4). The highest *n* − 1 stutter ratio was observed for the two three-base-pair Y-chromosome repeats *DYS481* and *DYS612* that both had an average stutter ratio of 31.5%, and a maximum stutter ratio of 43% and 37%, respectively. In contrast, the four five-base-pair repeat STR systems *Penta D*, *Penta E*, *DYS438* and *DYS643* had some of the lowest *n* − 1 stutter ratios, and no stutter was observed in the six-base-pair repeat system *DYS448*. At four STR loci, *D12S391*, *D21S11*, *DYS390* and *DXS7132*, two stutters of the same length but with different sequences were observed, and at *DYS389II*, three same-length stutters with different sequences were observed. These STRs have two or more sub-repeats, and slippage during the PCR may occur at any of the sub-repeats and, thus, generate stutter artefacts. For example, the allele *D21S11*_27_[TCTA]_6_[itvsTCTA]_8_ (itvs: the intervening sequence [TCTG]_5_[TCTA]_3_[TA]_1_[TCTA]_3_[TCA]_1_[TCTA]_2_[TCCATA]_1_) generated the stutters *D21S11*_26_[TCTA]_6_[itvsTCTA]_7_ (stutter ratio: 3.4%) and *D21S11*_26_[TCTA]_5_[itvsTCTA]_8_ (stutter ratio: 3.2%). Similarly, the allele *D12S391*_21_[AGAT]_12_[AGAC]_9_ generated the two stutters *D12S391*_20_[AGAT]_11_[AGAC]_9_ (stutter ratio: 17.4%) and *D12S391*_20_[AGAT]_12_[AGAC]_8_ (stutter ratio: 3.0%). In general, the fraction of stutter reads depended on the sequence and the length of the longest uninterrupted repeat [31,41–43]. The stutter ratio was high for alleles with long uninterrupted repeats. This was seen in e.g. *D18S51*, where the stutter ratio was linearly correlated with the number of [AGAA] repeats (Figure 3(A)). STR alleles with microvariants in the longest repeat had lower stutter ratios than alleles of similar length without microvariants, e.g. *D1S1656* (compare e.g. alleles 16.3 and 17 in Figure 3(B)). *D1S1656* had a long sequence of [TCTA] repeats. However, in some individuals, a [TGA] trinucleotide broke the longest repeat up into two smaller repeats. These alleles had much lower stutter ratios than alleles without the [TGA] sequence. In contrast, microvariants in *D19S433* did not affect the stutter ratio because the variants did not interrupt the longest repeat (compare e.g. alleles 14.2 and 15 in Figure 3(C)). Similarly, variations in the sub-repeat structures caused stutter ratios to be lower than the expected ones from the total number of repeats. *D2S1338* consisted of a long stretch of [TTCC] repeats. However, a single [GTCC] sequence interrupted the stretch of [TTCC] repeats in alleles with 22 or more repeats. Correspondingly, lower stutter ratios were observed for the longest alleles (compare e.g. alleles 21 and 22 in Figure 3(D)).

**Table 3.** Average stutter/parent allele read percentage of the STR markers.

Marker^a^^,^^b^	Average stutter read fraction in per cent (range)	*n* − 1 Stutter threshold (%) in the ForenSeq™ UAS
*Autosomal STRs*		
*FGA*	15.1 (6.2–24.4)	25.0
*D8S1179*	14.5 (7.5–20.9)	25.0
*D12S391 A*	14.5 (3.1–24.8)	33.0
*D17S1301*	13.5 (8.7–20.3)	20.0
*D2S1338*	13.4 (7.6–19.5)	20.0
*D1S1656*	12.8 (6.0–26.4)	25.0
*D18S51*	12.1 (7.2–20.2)	22.0
*D20S482*	10.9 (6.8–16.1)	15.0
*vWA*	10.8 (5.0–18.1)	22.0
*D10S1248*	10.3 (5.3–14.4)	20.0
*D16S539*	9.9 (4.7–15.3)	20.0
*D3S1358*	8.7 (6.3–12.8)	15.0
*D6S1043*	8.4 (5.4–12.4)	12.5
*D22S1045*	8.0 (3.5–18.9)	20.0
*D9S1122*	8.0 (3.4–13.7)	12.5
*D19S433*	7.7 (3.9–15.8)	12.5
*D21S11 A*	6.9 (3.1–11.7)	10.0
*D5S818*	6.2 (3.3–13.8)	12.5
*TH01*	6.1 (2.8–8.9)	10.0
*D7S820*	5.8 (3.5–9.9)	10.0
*Penta E*	5.2 (2.0–12.3)	10.0
*TPOX*	4.1 (1.9–5.9)	10.0
*D13S317*	3.6 (1.8–5.1)	12.5
*CSF1PO*	3.6 (2.0–6.9)	10.0
*D21S11 B*	3.5 (1.6–5.2)	10.0
*D4S2408*	3.3 (1.7–5.6)	7.5
*D12S391 B*	3.2 (1.4–4.4)	33.0
*D2S441*	3.0 (1.7–4.4)	7.5
*Penta D*	2.2 (1.1–4.1)	7.5
*Y-STRs***^c^**		
*DYS481*	31.5 (25.8–43.0)	50.0
*DYS612*	31.5 (23.6–36.8)	35.0
*DYS385a/b*	16.9 (3.8–31.8)	20.0
*DYS392*	14.8 (3.9–52.5)	30.0
*DYS570*	13.9 (11.0–19.8)	22.0
*DYF387S1*	12.1 (6.7–21.5)	20.0
*DYS389II A*	11.7 (5.6–17.1)	35.0
*DYS576*	10.9 (8.5–13.6)	15.0
*DYS389I*	10.4 (1.9–13.0)	20.0
*DYS391*	10.2 (7.3–12.2)	20.0
*Y-GATA-H4*	9.1 (6.4–13.2)	35.0
*DYS19*	7.3 (3.4–9.4)	15.0
*DYS390 A*	6.9 (5.0–11.0)	15.0
*DYS437*	6.7 (5.0–9.3)	45.0
*DYS389II B*	6.5 (2.0–13.1)	35.0
*DYS505*	6.5 (4.4–9.1)	15.0
*DYS460*	6.3 (4.6–8.0)	15.0
*DYS635*	5.8 (2.7–8.0)	15.0
*DYS533*	5.6 (4.2–7.7)	15.0
*DYS439*	4.9 (3.3–6.8)	15.0
*DYS522*	4.8 (2.3–10.0)	15.0
*DYS389II C*	4.2 (2.7–6.8)	35.0
*DYS549*	3.8 (2.6–5.5)	22.0
*DYS643*	3.0 (1.4–5.3)	20.0
*DYS390 B*	2.0 (1.4–3.0)	15.0
*DYS438*	1.7 (1.3–2.3)	15.0
*DYS448*	No stutters observed	15.0
*X-STRs*		
*DXS7132 A*	13.2 (2.9–18.6)	22.0
*DXS10135*	12.9 (7.4–33.7)	22.0
*DXS10103*	10.3 (2.9–26.7)	22.0
*DXS8378*	6.8 (3.0–14.2)	15.0
*DXS10074*	5.5 (1.8–9.4)	25.0
*DXS7423*	3.8 (2.5–6.1)	15.0
*HPRTB*	3.6 (2.1–5.7)	15.0
*DXS7132 B*	2.5 (2.5–2.5)	22.0

^a^Two different stutters with the same length were observed for *D12S391* (26 times), *DYS390* (24 times), *DYS389II* (24 times), *D21S11* (10 times) and *DXS7132* (1 time). The one with the most reads was designated A, and the one with the least reads was designated B; ^b^Three different stutters with the same length were observed in *DYS389II* (6 times). The one with the most reads was designated A, the one with the second most reads was designated B and the one with the least reads was designated C; ^c^Stutter percentages were not assessed for *DYS461*.

Only stutter reads that were one repeat unit shorter than the parent allele were considered here. Many of the sequences that were two repeats shorter (*n* − 2 stutter) or one repeat longer (*n* + 1 stutter) than the parent allele were not represented in the STRinNGS output file because STRinNGS ignored unique sequences with less than 1% of the total reads for a locus. Nevertheless, *n* − 2 and *n* + 1 stutters were observed in loci with frequent *n* − 1 stutter reads, e.g. *DYS481*, *DYS612*, *DYS385a/b*, *DYS392* and *DXS7132*.

### HID-SNP typing using the MiSeq FGx™ forensic genomics system

A total of 94 HID-SNPs were amplified with the ForenSeq™ DNA Signature Prep Kit, and analysed with the ForenSeq™ UAS. Eighteen locus drop-outs (0.32%) were observed in the duplicate typing of the 30 samples. Eleven of the 18 locus drop-outs were found at the rs1736442 SNP. The remaining locus drop-outs were detected in rs1031825, rs2920816, rs907100 and rs7041158.

A total of 2820 SNPs were typed twice, and 2780 genotypes (98.6%) were reproduced. The inconsistencies were caused by the 18 locus drop-outs mentioned above, 21 allele drop-outs at 8 loci (rs2920816, rs1493232, rs1031825, rs1294331, rs7041158, rs1736442, rs1454361 and rs338882) and one allele drop-in at rs1454361. Most allele drop-outs were found in rs2920816 and rs1493232 with seven and four incidents, respectively. Allele drop-outs were observed in loci with low read depth (<94×). The locus drop-in occurred in a locus with high read depth (846×) and very skewed Hb (24.6).

### Analysis of SNP amplicons

The locus balance, Hb and noise level of each of the 172 SNP loci were analysed using the freeware GATK (https://software.broadinstitute.org/gatk/) and the fastq files from the duplicate typing of 30 samples. The median read depth was 505 reads or around six times lower than that of the STR loci. The median read depth ranged from 60 (rs1736442) to 1 212 (rs917115) reads (Supplementary Figure 1). Seventeen of the 18 locus drop-outs and 13 of the 21 allele drop-outs were observed in the four loci with the lowest overall read depth (rs1736442, rs2920816, rs1031825 and rs7041158). A read depth of 200× has previously been suggested as a threshold for SNP typing with NGS platforms in relationship testing [8]. Eighty-six of the HID SNPs (91%), 50 of the AIM SNPs (93%) and all phenotypical SNPs had median read depth higher than 200×.

Hb (Supplementary Figure 2) was calculated for SNP loci with six or more heterozygous genotype calls among the duplicate sequencing of 30 individuals (150 loci). For most loci, the median Hb was close to one, and there were only few outliers. However, four SNPs (rs798443, rs338882, rs6955448 and rs279844) had median Hbs below 0.5 or higher than 2.0.

The reads that differed from the SNP genotype call were designated as noise. Only two SNP loci, rs1229984 and rs1979255, had median noise levels >0.1%. Of the 10 302 SNP genotypes, only 46 (0.45%) had noise levels >1%. Almost 50% (20) were at the rs1979255 locus.

### Sensitivity

**Figure 4.**
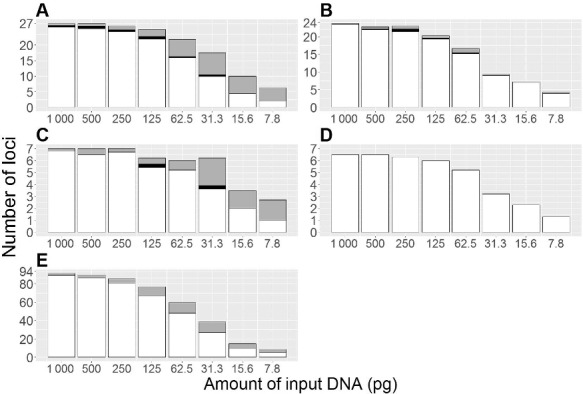
Sensitivity study. DNA extracts from two males and two females were serially diluted and typed in triplicate. The amount of DNA in the first PCR was 1 000, 500, 250, 125, 62.5, 31.3, 15.6 and 7.8 pg, respectively. The average number of correctly typed loci (white), loci typed with allele drop-in (black) and loci typed with allele drop-out (grey) is shown for autosomal STRs (A: 27 loci), Y-STRs (B: 24 loci), X-STRs in female samples (C: 7 loci), X-STRs in male samples (D: 7 loci) and HID-SNPs (E: 94 loci). Two different amplicons were generated from each of the loci: *DYS385a/b* and *DYF387S1*. Y-STR allele drop-out refers to the drop-out of one of these alleles.

Eight dilutions of DNA from two male and two female individuals were amplified in triplicates using Primer Mix A and genotyped with the MiSeq FGx™ Forensic Genomics System (Pool 3). Overall, the typing efficiency and quality of the results decreased with decreasing amount of DNA in the first PCR. The number of allele and locus drop-outs increased dramatically when the DNA input was <250 pg (Figure 4). Similarly, the number of loci flagged for low read depth by the ForenSeq™ UAS increased dramatically for <250 pg input DNA (Table 4).

**Table 4.** Number of loci assigned a warning by the ForenSeq™ UAS for low read depth or imbalance^a^.

	Autosomal STRs (27)^b^		Y-STRs (24)^b^	X-STRs (7)^b^		HID SNPs (94)^b^	
Input DNA (pg)	Low read depth	Imbalance	Low read depth	Low read depth	Imbalance	Low read depth	Imbalance
1 000.0	0.2	1.9	0.3	0.3	0.5	2.7	1.8
500.0	0.1	3.7	1.3	0.3	0.3	3.9	3.2
250.0	0.8	5.4	1.0	0.3	2.0	8.3	3.0
125.0	2.0	7.5	3.5	0.9	2.0	17.1	4.5
62.5	5.3	5.3	7.2	1.4	1.8	34.5	2.3
31.3	9.5	2.6	14.0	2.3	1.5	55.4	1.1
15.6	17.0	0.8	15.8	4.1	0.2	79.3	0.3
7.8	20.8	0.1	19.0	5.0	0.2	85.6	0.0

a The averages of triplicate typing of four samples (two male and two female samples); ^b^The total number of loci is shown in parentheses.

*DXS10103* and *DYS389II* were the STR markers most prone to locus drop-out with ≥250 pg input DNA (locus drop-outs occurred in 56% and 39% of the male samples, respectively; see Supplementary Table 1). *DXS10103* had a lower read depth than the other loci (Figure 1). The high susceptibility to locus drop-out of *DYS389II* may be due to the long alleles of this locus (http://strbase.nist.gov/str_y389.htm). HID SNPs prone to locus drop-outs with ≥250 pg input DNA included the four SNP loci with the lowest read depth, rs2920816, rs1031825, rs7041158 and rs1736442 (Supplementary Figure 1), as well as rs354439 and rs1294331.

High proportions of allele drop-out with ≥250 pg input DNA were observed for *DYS385a/b*, *DXS10103*, *vWA* and *D1S1656* (Supplementary Table 2). *D1S1656* was the marker with the largest variation in Hb. The *DYS385a/b* amplicons were long (≥316 bp) [44] including a 163 bp upstream flanking sequence. *DXS10103* and *vWA* were among the loci with the lowest read depth (Figure 1 and Table 2). HID SNP loci prone to allele drop-outs with ≥250 pg input DNA included rs1493232 and rs10488710 (allele drop-out in 28% and 22% of the samples, respectively). Both loci frequently experienced locus drop-outs at <250 pg input DNA.

Sequences with *n* − 1 repeats (most likely *n* − 1 stutters) that had high read counts led to allele drop-ins. At ≥250 pg input DNA, allele drop-ins were observed in *DYS612*, *D20S482*, *D9S1122*, *D2S1338*, *D3S1358*, *FGA*, *DYS505*, *D6S1043*, *D8S1179*, *TH01* and *D21S11* (Supplementary Table 3). No allele drop-in was observed in the HID SNP loci.

### DNA mixture detection

DNA from a male and a female was mixed in 15 different ratios, and sequenced with the MiSeq FGx™ Forensic Genomics System in duplicate (Pool 4). The ForenSeq™ UAS provides tools for identification of mixtures. For each locus, alerts for low quality are raised if the Hb is too skewed or if the number of detected alleles exceeds two (for STRs). The “single-source indicator” calls the sample as single-source if the number of violations of the intralocus balance threshold and allele count is <6 for STRs, and/or if the number of violations of the intralocus balance threshold is <11 for HID SNPs (AIMs and phenotypical SNPs are not used to indicate whether the sample is a mixture). The results from the ForenSeq™ UAS analysis of the 30 mixtures are shown in Table 5. All samples with mixture ratios between 1:1 and 1:50 were identified as mixtures, whereas 1:100 and 1:1 000 mixtures were called as single-source samples. It is noteworthy that Y-STR alleles were detected in 1:100 (six and seven alleles in the two duplicates) and 1:1000 (two and zero alleles) male/female mixtures, and that this did not influence the single-source indicator of the ForenSeq™ UAS (data not shown).

**Table 5.** Analysis of mixtures with the ForenSeq™ UAS.

		Autosomal STRs		HID SNPs
Mixture ratio (male/female)	“Single source” indicator^a^	Average number of “allele count” alerts^b^	Average number of “imbalance” alerts^b^	Average number of “imbalance” alerts^b^
1 000:1	Pass	1.5	0.5	3.5
100:1	Pass	3	3	1.5
50:1	Fail	8.5	3	2
25:1	Fail	9.5	4.5	6
12:1	Fail	16	9	12.5
6:1	Fail	19	19	32.5
3:1	Fail	19	20	48.5
1:1	Fail	20	17.5	45.5
1:3	Fail	19	21	29.5
1:6	Fail	17	19.5	18
1:12	Fail	14.5	13.5	10.5
1:25	Fail	10	5	4.5
1:50	Fail	4.5	2.5	3.5
1:100	Pass	1	1	2.5
1:1 000	Pass	1	2.5	2.5
Single contributor samples^c^	Pass	0.5	2.2	1.05

a “Pass” indicates that the DNA originates from only one source, while “Fail” indicates that the sample contains DNA from more than one individual; ^b^Average of duplicate typing; ^c^Results from 30 samples typed in duplicate (Pools 1 and 2).

The average numbers of alerts for the 30 single-source samples typed in duplicate (Pools 1 and 2) were 2.7 and 1.05 for STRs and SNPs, respectively (Table 5). The “allele count” alerts were seen in *D7S820* (eight times), *D20S482* (six times) and *D21S11* (five times). These loci had a relatively low stutter threshold compared to the observed fraction of stutter reads (Table 3). Sometimes, the number of *n* − 1 stutter reads exceeded the threshold, which released an “allele count” alert. The largest number of alerts for “imbalance” was observed in *D22S1045* (44 times), *D1S1656* (26 times), *Penta E* (12 times), *D5S818* (11 times), rs338882 (19 times), rs6955448 (12 times) and rs4530059 (9 times). These loci have either skewed median Hb or large variations of Hb (Figure 2, Supplementary Figure 2), which triggered the “imbalance” alert of some samples.

### Ancestry prediction

The ForenSeq™ UAS comprises a tool for prediction of biogeographic ancestry using four reference populations: European, East Asian, African and admixed American. The predictions were compared to self-reported ancestry of 30 individuals. Twenty out of 21 self-reported Europeans were assigned European ancestry. One of the Italian individuals was predicted as admixed American. The two self-reported East Asians (Korean and Chinese) were assigned East Asian ancestry. Two Africans from Ethiopia were predicted as African and admixed American, respectively. The Brazilian individual was predicted to be of admixed American ancestry, and the Argentinian individual to be of European ancestry. The Argentinian individual had blue eyes and blond hair (see the following paragraph) indicating that the individual was of European descent. Finally, a Moroccan, an Iraqi and an Indian individual were all assigned as admixed Americans.

### Phenotype prediction

The ForenSeq™ UAS comprises a tool for prediction of eye and hair colours based on the HIrisplex model [25]. The eye colour predictions were compared to the PIE-scores that are an objective measure of the eye colour [40], whereas the hair colour predictions were compared to self-reported hair colours.

**Figure 5.**
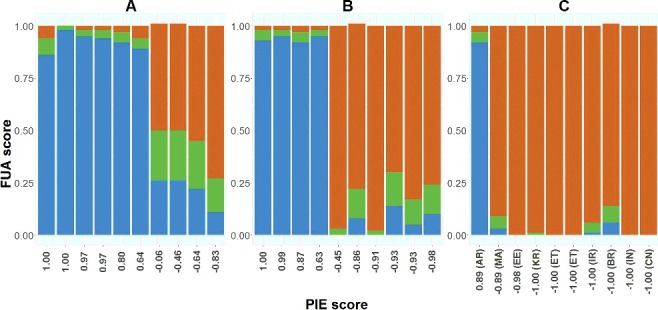
Correlation between the objective PIE-score eye colour measure and eye colour predictions obtained with ForenSeq™ UAS in 30 individuals: 10 Danes (A), 10 Italians (B) and 10 individuals of other origin (AR: Argentina, MA: Morocco, EE: Eastern Europe, KR: Korea, ET: Ethiopia, IR: Iraq, BR: Brazil, IN: India and CN: China) (C). Blue, green and brown colours indicate the scores for blue, intermediate and brown eye colours, respectively.

The ForenSeq™ UAS reported probability values for each colour category. With a probability threshold of *P* > 0.7, as suggested in [25], 11 of the 30 individuals were predicted to have blue eyes, 16 individuals to have brown eyes and the eye colours of 3 individuals could not be predicted (Figure 5). Of the 11 individuals predicted to have blue eyes, 9 had PIE-scores >0.8, and 2 individuals had PIE-scores of 0.63 and 0.64, respectively. Most likely, the eyes of individuals with PIE-scores >0.8 would be categorized as blue or light-coloured, whereas PIE-scores around 0.6 most likely are associated with an intermediate eye colour [40].

**Figure 6.**
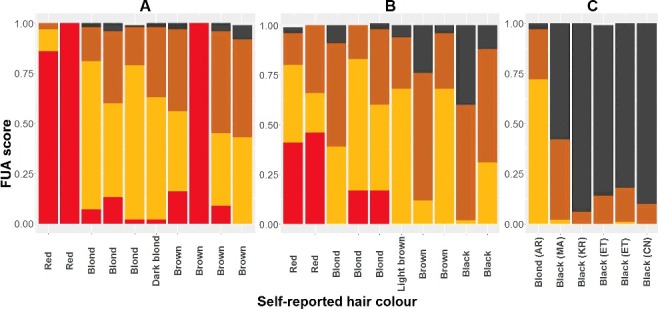
Comparison between self-reported hair colour and hair colour predictions obtained with ForenSeq™ UAS in 26 individuals: 10 Danes (A), 10 Italians (B) and 6 individuals of other origin (C) (AR: Argentina, MA: Morocco, KR: Korea, ET: Ethiopia and CN: China). Red, blond, brown and black colours indicate the probability values of red, blond, brown and black hair colours, respectively.

With a probability threshold of *P* > 0.7, the hair colour was predicted for only 10 of the 30 individuals (Figure 6). Nine of the predictions were in concordance with the self-reported hair colour including two, three and four individuals with red, blond and black hair colour, respectively. One individual was predicted to have red hair, but had brown hair colour. Noteworthy, no brown hair colour predictions were made, even though seven individuals had self-reported brown hair colour.

## Discussion

The MiSeq FGx™ Forensic Genomics System [11] is capable of typing 153 (Panel A) or 231 (Panel B) loci commonly used in forensic genetics in one multiplex assay. The loci include STRs and SNPs, which make the ForenSeq™ DNA Signature Prep Kit the first commercial assay for typing of both types of HID markers and an excellent example of what PCR–NGS assays may provide to forensic genetic investigations in future.

Except for two drop-ins in *D21S11* (Supplementary File 3), complete concordance between the automated ForenSeq™ UAS analysis and the PCR–CE results was observed. Both drop-ins were flagged by the ForenSeq™ UAS, and would have been called as stutter artefacts by the data analyst. The reproducibility was >98.5% for both STRs and SNPs in the duplicate typing of 30 samples. Most of the discordances were caused by locus or allele drop-outs, whereas allele drop-ins were rare and mainly caused by stutter artefacts. The analysis of the assay sensitivity indicated that most loci may be successfully typed with an input DNA amount of >250 pg in the first PCR. In the reproducibility and sensitivity studies, *DXS10103* and the four HID-SNPs, rs1736442, rs2920816, rs1031825 and rs7041158, accounted for the majority of drop-outs. All these loci had relatively low read counts (Figure 1 and Supplementary Figure 1), which explained why they frequently dropped-out. Analysis of the heterozygote balances revealed that *D22S1045* was another poorly performing locus. The variation in allele read depth was so large that allele drop-out was likely, and it was difficult to distinguish stutters from true alleles for some samples. The power of discrimination of the MiSeq FGx™ Forensic Genomics System is so large that it may be considered to eliminate these loci from the assay. However, *D22S1045* is one of the ESS markers. Therefore, it would be preferable to keep the locus in the multiplex, but design new, more efficient PCR primers. Similarly, one of the YHRD core loci [23], *DYS393*, is not amplified with the MiSeq FGx™ Forensic Genomics System, and it should be included in any future design.

The ForenSeq™ UAS is not an expert software that reports genotypes and conclusions in a final report without manual inspection of a case officer. The software assists the data analyst by presenting the analysed results in detailed reports and by raising alerts, but it is the data analyst who makes the conclusions. The software uses two minimal thresholds: the IT and the analytical threshold (AT). If the locus read depth is less than 650 reads, the IT is set to 30 reads, and the AT to 10 reads. When the read depth is higher, the software employs IT and AT settings that are user-defined as percentages of the total reads for each locus. Only sequences with read depth higher than the IT are called. The AT is used exclusively to raise alerts and to inform the data analyst that there are sequences with fewer reads, which may be relevant for the interpretation. The threshold for the Hb is also user-defined. However, it is set commonly for all STRs and all SNPs, and not for each locus, separately. Some loci have skewed Hbs (Figure 2 and Supplementary Figure 2) and frequently trigger “imbalance” alerts. Therefore, it would be convenient if locus-specific Hb thresholds were implemented in future versions of the software. The ForenSeq™ UAS uses the number of “imbalance” and “allele count” alerts to identify possible mixtures. However, it is up to the data analyst to act on this information and draw the necessary conclusions. The locus-specific stutter filters are user-defined and take the possibility of observing *n* − 1, *n* − 2 and *n* + 1 stutters into account. The filter for each locus is a fixed percentage of the number of reads of the parent allele (Table 3). However, as shown in Figure 3 and Supplementary File 4, the stutter ratio varied between alleles of the same locus and depended on the longest uninterrupted repeat [31,41,42]. In the ideal situation, the stutter filter values should be set for each allele, i.e. be sequence-dependent.

In this work, STRinNGS [5] was used to verify the data analysis of the ForenSeq™ UAS and to analyse the flanking regions of the STRs. We found true sequence variations in the flanking regions (all previously reported by Novroski et al. [6]), and what appeared to be sequencing errors or base degeneration in the primer sets of the first PCR. The latter were problematic because of their high frequency at some loci. For example, in *D1S1656*, four unique sequences with almost equal read counts in all heterozygous individuals were identified: two different STR alleles and for each STR allele, two different SNP–STR alleles. The variant in the flanking region of *D1S1656* was detected 6 bp downstream and was most likely caused by a degenerated base in the downstream PCR primer. These positions must be identified to allow proper analysis of the flanking regions and should be made available by the manufacturer (Illumina or Verogen). The ForenSeq™ UAS does not report variants in the flanking regions, except for the SNP rs73801920 positioned 4 bp downstream of *D5S818* [5]. Variation in this position is indicated in the report, if an individual has two SNP–STR alleles of the same size. However, it is up to the data analyst to use that information and name the SNP–STR alleles. In the case of an insertion or deletion in the flanking region, the ForenSeq™ UAS reports the allele name based on the length of the fragment, similarly to PCR–CE detection methods, and the sequence of the STR region as a string of bases. Again, it is up to the data analyst to interpret the STR sequence and name the allele. Two loci with deletions in the flanking regions were identified in our study. One individual had a 13 bp deletion immediately upstream of *Penta D*, and four individuals had a 3 bp deletion in the downstream flank of *DXS10135* (Table 6). In the example with a deletion in *Penta D*, the ForenSeq™ UAS named the allele *Penta D*[2.2], which is identical to the PCR–CE allele name. However, the allele had five [AAAGA] repeats, and the 13 bp deletion was not reported. Without a more detailed analysis of the reads, it was not possible for the data analyst to give the allele the accurate name, which should be *Penta D*_2.2_[AAAGA]_5_del:45056073-85 [36]. Similarly, the deletion at *DXS10135* was not reported by the ForenSeq™ UAS, and the true name could only be constructed using another software.

**Table 6.** *Penta D* and *DXS10135* alleles with deletions in the STR flanking regions.

Locus	Number of repeats	CE allele name	Sequencing allele name^a^
*Penta D*	5	*Penta D*[2.2]	*Penta D*[CE2.2]-Chr21-GRCh37 45056085-45056150 [AAAGA]_5_ del:45056073-85
*DXS10135*	18	*DXS10135*[17.1]	*DXS10135*[CE17.1]-ChrX-GRCh37 9306342-9306440 [AAGA]_3_GAAAGGA[AAGA]_14_[AAAG] del:9306454-6
	20	*DXS10135*[19.1]	*DXS10135*[CE19.1]-ChrX-GRCh37 9306342-9306440 [AAGA]_3_GAAAGGA[AAGA]_16_[AAAG] del:9306454-6
	21	*DXS10135*[20.1]	*DXS10135*[CE20.1]-ChrX-GRCh37 9306342-9306440 [AAGA]_3_GAAAGGA[AAGA]_17_[AAAG] del:9306454-6
	22	*DXS10135*[21.1]	*DXS10135*[CE21.1]-ChrX-GRCh37 9306342-9306440 [AAGA]_3_GAAAGGA[AAGA]_18_[AAAG] del:9306454-6

a According to Parson et al. [35].

Overall, the performance of the MiSeq FGx™ Forensic Genomics System was high and the assay may be an attractive alternative to PCR–CE assays in some cases. In previous studies, other researchers reached similar conclusions on the overall performance of the MiSeq FGx™ Forensic Genomics System, and also identified the same error-prone loci [6,26–29]. However, the higher costs of the workflow compared to that of PCR–CE typing may limit its use to special cases. The reagent costs of typing a sample with the MiSeq FGx™ Forensic Genomics System was approximately US$ 60 with Primer Mix A, and US$ 90 with Primer Mix B. This is 3–4 times the price of PCR–CE typing of CODIS and ESS STR loci, which usually provides sufficient discrimination power for non-degraded single contributor samples [1,45]. In the analysis of mixtures and/or samples with low amounts of DNA, the STR sequence information and sheer number of typed loci may be valuable. However, the high stutter ratios of some of the loci may interfere with mixture interpretation (Table 3, Supplementary File 4). Ancestry and phenotype information may provide investigative leads to the police in crime cases without a suspect and no hit in the crime DNA database, as well as in cases with missing persons [25,40,46]. However, the ancestry and phenotype prediction provided by the MiSeq FGx™ Forensic Genomics System was not based on the likelihood ratio principle, which is the internationally recommended method for reporting forensic genetic evidence [10,47,48]. This should also be addressed in future versions of the ForenSeq™ UAS.

## Compliance with ethical standards

All procedures performed in studies involving human participants were in accordance with the ethical standards of the Danish Ethical Committee (H-3-2012-023) and with the 1964 Helsinki declaration and its later amendments or comparable ethical standards. Samples were taken from the biobank of the Department of Forensic Medicine, University of Copenhagen (RIBVF; approved by the Danish Data Protection Agency, j.no. 2002-54-1080). The Danish ethical committee waived the requirement for informed consent (H-3-2012-023).

## Supplementary Material

Supp_mat_1446672_TFSR.zip

## References

[1] Gill P, Haned H, Bleka O, et al. Genotyping and interpretation of STR-DNA: low-template, mixtures and database matches – twenty years of research and development. Forensic Sci Int Genet. 2015;18:100–117. 25866376 10.1016/j.fsigen.2015.03.014

[2] Fordyce SL, Avila-Arcos MC, Rockenbauer E, et al. High-throughput sequencing of core STR loci for forensic genetic investigations using the Roche Genome Sequencer FLX platform. Bio Techniques. 2011;51:127–133. 10.2144/000113721 21806557

[3] Fordyce SL, Mogensen HS, Borsting C, et al. Second-generation sequencing of forensic STRs using the ion torrent HID STR 10-plex and the ion PGM. Forensic Sci Int Genet. 2015;14:132–140. 25450784 10.1016/j.fsigen.2014.09.020

[4] Borsting C, Morling N Next generation sequencing and its applications in forensic genetics. Forensic Sci Int Genet. 2015;18:78–89. 25704953 10.1016/j.fsigen.2015.02.002

[5] Friis SL, Buchard A, Rockenbauer E, et al. Introduction of the Python script STRinNGS for analysis of STR regions in FASTQ or BAM files and expansion of the Danish STR sequence database to 11 STRs. Forensic Sci Int Genet. 2016;21:68–75. 26722765 10.1016/j.fsigen.2015.12.006

[6] Novroski NM, King JL, Churchill JD, et al. Characterization of genetic sequence variation of 58 STR loci in four major population groups. Forensic Sci Int Genet. 2016;25:214–226. 27697609 10.1016/j.fsigen.2016.09.007

[7] Grandell I, Samara R, Tillmar AO A SNP panel for identity and kinship testing using massive parallel sequencing. Int J Legal Med. 2016;130:905–914. 26932869 10.1007/s00414-016-1341-4

[8] Buchard A, Kampmann ML, Poulsen L, et al. ISO 17025 validation of a next-generation sequencing assay for relationship testing. Electrophoresis. 2016;37:2822–2831. 27709635 10.1002/elps.201600269

[9] van der Gaag KJ, de Leeuw RH, Hoogenboom J, et al. Massively parallel sequencing of short tandem repeats – population data and mixture analysis results for the PowerSeq system. Forensic Sci Int Genet. 2016;24:86–96. 27347657 10.1016/j.fsigen.2016.05.016

[10] Pereira V, Mogensen HS, Borsting C, et al. Evaluation of the Precision ID ancestry panel for crime case work: a SNP typing assay developed for typing of 165 ancestral informative markers. Forensic Sci Int Genet. 2017;28:138–145. 28273506 10.1016/j.fsigen.2017.02.013

[11] Jager AC, Alvarez ML, Davis CP, et al. Developmental validation of the MiSeq FGx forensic genomics system for targeted next generation sequencing in forensic DNA casework and database laboratories. Forensic Sci Int Genet. 2017;28:52–70. 28171784 10.1016/j.fsigen.2017.01.011

[12] Wang Z, Zhou D, Wang H, et al. Massively parallel sequencing of 32 forensic markers using the Precision ID GlobalFiler NGS STR Panel and the ion PGM system. Forensic Sci Int Genet. 2017;31:126–134. 28938153 10.1016/j.fsigen.2017.09.004

[13] van der Heijden S, de Oliveira SJ, Kampmann ML, et al. Comparison of manual and automated AmpliSeq workflows in the typing of a Somali population with the Precision ID Identity Panel. Forensic Sci Int Genet. 2017;31:118–125. 28938152 10.1016/j.fsigen.2017.09.009

[14] Mertens G, Gielis M, Mommers N, et al. Mutation of the repeat number of the HPRTB locus and structure of rare intermediate alleles. Int J Legal Med. 1999;112:192–194. 10335885 10.1007/s004140050231

[15] Edelmann J, Deichsel D, Hering S, et al. Sequence variation and allele nomenclature for the X-linked STRs DXS9895, DXS8378, DXS7132, DXS6800, DXS7133, GATA172D05, DXS7423 and DXS8377. Forensic Sci Int. 2002;129:99–103. 12243877 10.1016/s0379-0738(02)00230-x

[16] Becker D, Rodig H, Augustin C, et al. Population genetic evaluation of eight X-chromosomal short tandem repeat loci using Mentype Argus X-8 PCR amplification kit. Forensic Sci Int Genet. 2008;2:69–74. 19083792 10.1016/j.fsigen.2007.08.013

[17] Rodig H, Kloep F, Weissbach L, et al. Evaluation of seven X-chromosomal short tandem repeat loci located within the Xq26 region. Forensic Sci Int Genet. 2010;4:194–199. 20215031 10.1016/j.fsigen.2009.08.010

[18] Hering S, Augustin C, Edelmann J, et al. DXS10079, DXS10074 and DXS10075 are STRs located within a 280-kb region of Xq12 and provide stable haplotypes useful for complex kinship cases. Int J Legal Med. 2006;120:337–345. 16344967 10.1007/s00414-005-0061-y

[19] Ballantyne KN, Goedbloed M, Fang R, et al. Mutability of Y-chromosomal microsatellites: rates, characteristics, molecular bases, and forensic implications. Am J Hum Genet. 2010;87:341–353. 20817138 10.1016/j.ajhg.2010.08.006 PMC2933352

[20] Kidd KK, Pakstis AJ, Speed WC, et al. Developing a SNP panel for forensic identification of individuals. Forensic Sci Int. 2006;164:20–32. 16360294 10.1016/j.forsciint.2005.11.017

[21] Sanchez JJ, Phillips C, Borsting C, et al. A multiplex assay with 52 single nucleotide polymorphisms for human identification. Electrophoresis. 2006;27:1713–1724. 16586411 10.1002/elps.200500671

[22] Budowle B, Moretti TR, Baumstark AL, et al. Population data on the thirteen CODIS core short tandem repeat loci in African Americans, U.S. Caucasians, Hispanics, Bahamians, Jamaicans, and Trinidadians. J Forensic Sci. 1999;44:1277–1286. 10582369

[23] Roewer L, Krawczak M, Willuweit S, et al. Online reference database of European Y-chromosomal short tandem repeat (STR) haplotypes. Forensic Sci Int. 2001;118:106–113. 11311820 10.1016/s0379-0738(00)00478-3

[24] Kidd KK, Speed WC, Pakstis AJ, et al. Progress toward an efficient panel of SNPs for ancestry inference. Forensic Sci Int Genet. 2014;10:23–32. 24508742 10.1016/j.fsigen.2014.01.002

[25] Walsh S, Liu F, Wollstein A, et al. The HIrisPlex system for simultaneous prediction of hair and eye colour from DNA. Forensic Sci Int Genet. 2013;7:98–115. 22917817 10.1016/j.fsigen.2012.07.005

[26] Just RS, Moreno LI, Smerick JB, et al. Performance and concordance of the ForenSeq system for autosomal and Y chromosome short tandem repeat sequencing of reference-type specimens. Forensic Sci Int Genet. 2017;28:1–9. 28126691 10.1016/j.fsigen.2017.01.001

[27] Guo F, Yu J, Zhang L, et al. Massively parallel sequencing of forensic STRs and SNPs using the Illumina(R) ForenSeq DNA Signature Prep Kit on the MiSeq FGx forensic genomics system. Forensic Sci Int Genet. 2017;31:135–148. 28938154 10.1016/j.fsigen.2017.09.003

[28] Silvia AL, Shugarts N, Smith J A preliminary assessment of the ForenSeq FGx System: next generation sequencing of an STR and SNP multiplex. Int J Legal Med. 2017;131:73–86. 27785563 10.1007/s00414-016-1457-6

[29] Churchill JD, Novroski NMM, King JL, et al. Population and performance analyses of four major populations with Illumina's FGx Forensic Genomics System. Forensic Sci Int Genet. 2017;30:81–92. 28651097 10.1016/j.fsigen.2017.06.004

[30] Elwick K, Zeng X, King J, et al. Comparative tolerance of two massively parallel sequencing systems to common PCR inhibitors. Int J Legal Med. 2018 DOI:10.1007/s00414-017-1693-4 PMID: 28956146; eng. 28956146

[31] Tvedebrink T, Mogensen HS, Stene MC, et al. Performance of two 17 locus forensic identification STR kits – Applied Biosystems's AmpFℓSTR(R) NGMSElect and Promega's PowerPlex(R) ESI17 kits. Forensic Sci Int Genet. 2012;6:523–531. 22266064 10.1016/j.fsigen.2011.12.006

[32] Illumina: ForenSeq Universal Analysis Software Guide [Internet] 2016 Aug [cited 2017 Nov 17]. Available from: https://support.illumina.com/content/dam/illumina-support/documents/documentation/software_documentation/forenseq-universal-analysis-software/forenseq-universal-analysis-software-guide-15053876-01.pdf

[33] Gettings KB, Aponte RA, Vallone PM, et al. STR allele sequence variation: current knowledge and future issues. Forensic Sci Int Genet. 2015;18:118–130. 26197946 10.1016/j.fsigen.2015.06.005

[34] Zhu BF, Shen CM, Wang HD, et al. Genetic diversities of 21 non-CODIS autosomal STRs of a Chinese Tibetan ethnic minority group in Lhasa. Int J Legal Med. 2011;125:581–585. 21042917 10.1007/s00414-010-0519-4

[35] Parson W, Ballard D, Budowle B, et al. Massively parallel sequencing of forensic STRs: considerations of the DNA commission of the International Society for Forensic Genetics (ISFG) on minimal nomenclature requirements. Forensic Sci Int Genet. 2016;22:54–63. 26844919 10.1016/j.fsigen.2016.01.009

[36] Gelardi C, Rockenbauer E, Dalsgaard S, et al. Second generation sequencing of three STRs D3S1358, D12S391 and D21S11 in Danes and a new nomenclature for sequenced STR alleles. Forensic Sci Int Genet. 2014;12:38–41. 24893347 10.1016/j.fsigen.2014.04.016

[37] Schubert M, Lindgreen S, Orlando L AdapterRemoval v2: rapid adapter trimming, identification, and read merging. BMC Res Notes. 2016;9:88. 26868221 10.1186/s13104-016-1900-2 PMC4751634

[38] Li H, Handsaker B, Wysoker A, et al. The Sequence Alignment/Map format and SAMtools. Bioinformatics (Oxford, England). 2009;25:2078–2079. 10.1093/bioinformatics/btp352 PMC2723002 19505943

[39] Li H A statistical framework for SNP calling, mutation discovery, association mapping and population genetical parameter estimation from sequencing data. Bioinformatics (Oxford, England). 2011;27:2987–2993. 10.1093/bioinformatics/btr509 PMC3198575 21903627

[40] Andersen JD, Johansen P, Harder S, et al. Genetic analyses of the human eye colours using a novel objective method for eye colour classification. Forensic Sci Int Genet. 2013;7:508–515. 23948321 10.1016/j.fsigen.2013.05.003

[41] Hill CR, Duewer DL, Kline MC, et al. Concordance and population studies along with stutter and peak height ratio analysis for the PowerPlex (R) ESX 17 and ESI 17 Systems. Forensic Sci Int Genet. 2011;5:269–275. 20457109 10.1016/j.fsigen.2010.03.014

[42] Brookes C, Bright JA, Harbison S, et al. Characterising stutter in forensic STR multiplexes. Forensic Sci Int Genet. 2012;6:58–63. 21388903 10.1016/j.fsigen.2011.02.001

[43] Woerner AE, King JL, Budowle B Flanking variation influences rates of stutter in simple repeats. Genes. 2017;8(11):329. 10.3390/genes8110329 PMC5704242 29149052

[44] Illumina: ForenSeq™ DNA Signature Prep Guide [Internet]. 2015 Sep [cited 2017 Nov 17]. Available from: https://support.illumina.com/content/dam/illumina-support/documents/documentation/chemistry_documentation/forenseq/forenseq-dna-signature-prep-guide-15049528-01.pdf

[45] Jobling MA, Gill P Encoded evidence: DNA in forensic analysis. Nat Rev Genet. 2004;5:739–751. DOI:10.1038/nrg1455. 15510165

[46] Phillips C Forensic genetic analysis of bio-geographical ancestry. Forensic Sci Int Genet. 2015;18:49–65. 26013312 10.1016/j.fsigen.2015.05.012

[47] Morling N, Allen RW, Carracedo A, et al. Paternity testing commission of the international society of forensic genetics: recommendations on genetic investigations in paternity cases. Forensic Sci Int. 2002;129:148–157. 12372685 10.1016/s0379-0738(02)00289-x

[48] Gill P, Brenner CH, Buckleton JS, et al. DNA commission of the international society of forensic genetics: recommendations on the interpretation of mixtures. Forensic Sci Int. 2006;160:90–101. 16750605 10.1016/j.forsciint.2006.04.009

