# Supplementary material for: Sequencing of 231 forensic genetic markers using the MiSeq FGx™ forensic genomics system – an evaluation of the assay and software
Source: Forensic Sci Res. 2018 Apr 9;3(2):111–23. doi: 10.1080/20961790.2018.1446672 (PMC6197110; doi:10.1080/20961790.2018.1446672)
Supplement: Supp_mat_1446672_TFSR.zip [file TFSR_A_1446672_SM7836.zip › Supp_mat_1446672_TFSR/SupplFig2_SNPs_Hb.docx]

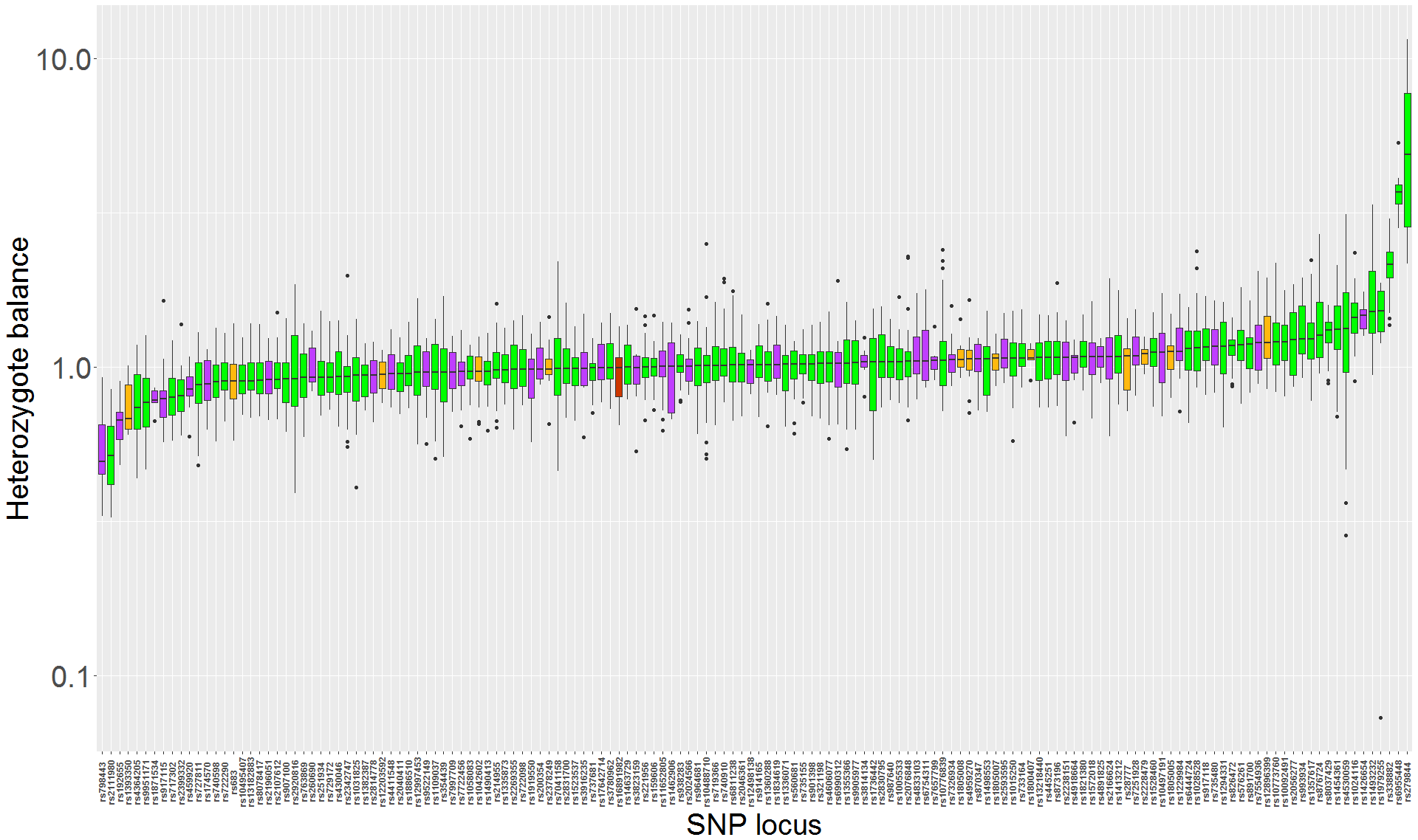


Supplementary Figure 2. Heterozygote balances of SNPs included in the MiSeq FGx^TM^ Forensic Genomics System. Only loci with six or more observed heterozygous individuals were included. HID-SNPs are marked with green colour, AIM SNPs with purple colour, phenotype-informative SNPs with yellow colour, and SNPs that are informative for both ancestry and phenotypes with red colour. The box-and-whisker plot parameters are described in Figure 1.
