# Supplementary material for: Sequencing of 231 forensic genetic markers using the MiSeq FGx™ forensic genomics system – an evaluation of the assay and software
Source: Forensic Sci Res. 2018 Apr 9;3(2):111–23. doi: 10.1080/20961790.2018.1446672 (PMC6197110; doi:10.1080/20961790.2018.1446672)
Supplement: Supp_mat_1446672_TFSR.zip [file TFSR_A_1446672_SM7836.zip › Supp_mat_1446672_TFSR/SupplFile4_all.pdf]

Supplementary File 4. Fractions of stutter reads compared to repeat lengths. The stutter ratio was calculated as the number of reads of the n-1 stutters divided by the number of reads of the parent alleles.

Autosomal STRs [a]

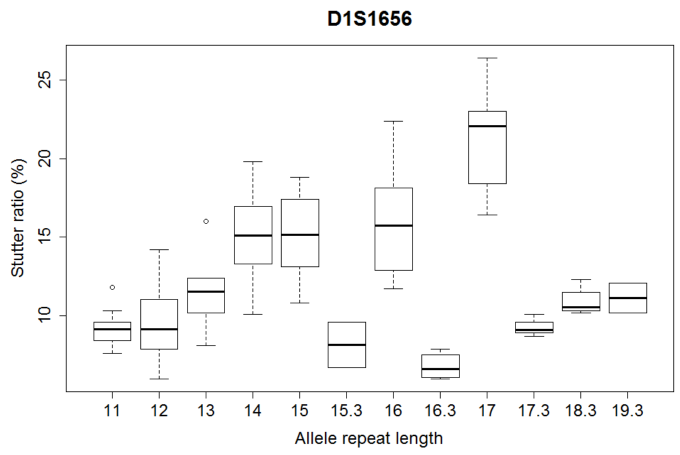

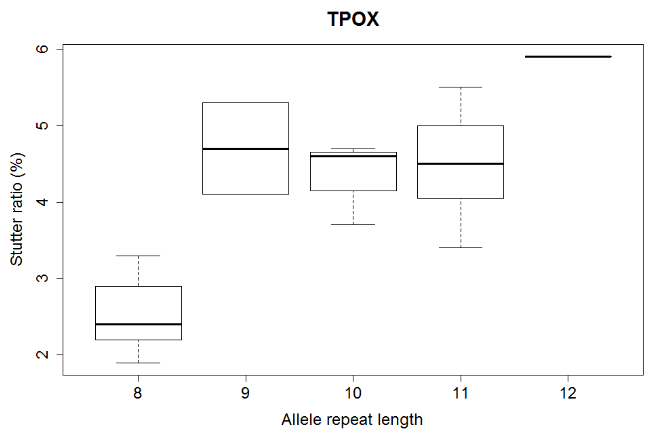

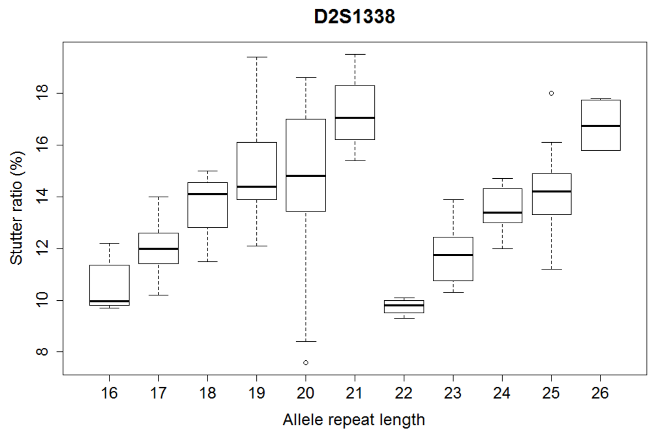

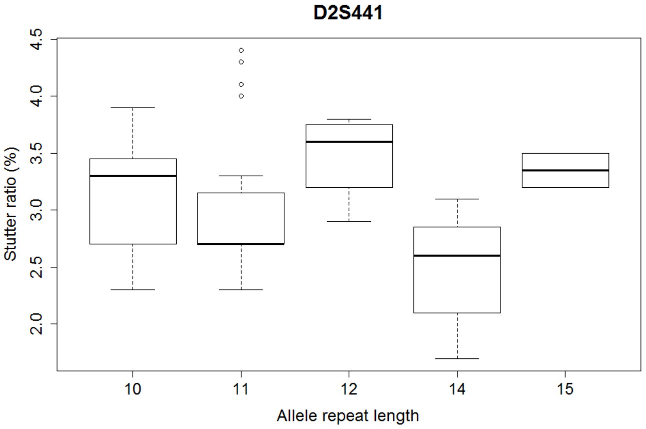
D2S441
Stutter ratio (%)
2.0
2.5
3.0
3.5
4.0
4.5
10
11
12
14
15
Allele repeat length

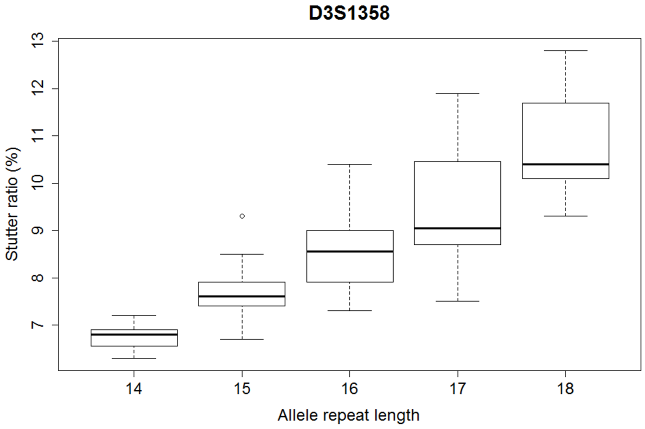
D3S1358
Stutter ratio (%)
7
8
9
10
11
12
13
14
15
16
17
18
Allele repeat length

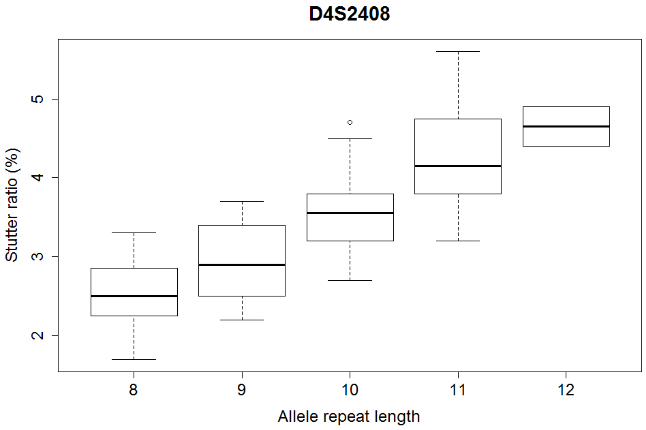
D4S2408
Stutter ratio (%)
2
3
4
5
8
9
10
11
12
Allele repeat length

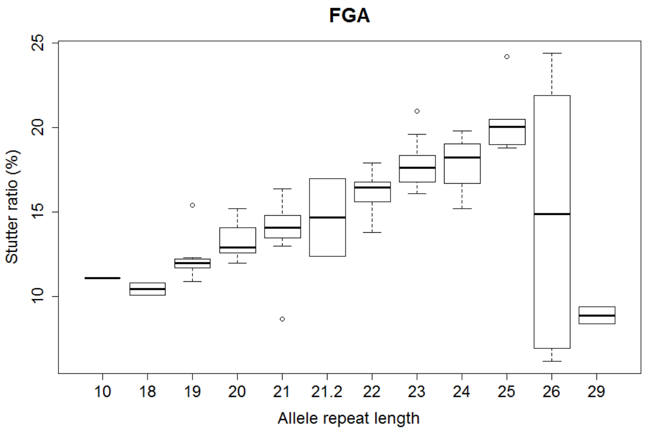
FGA
Stutter ratio (%)
10
15
20
25
10
18
19
20
21
21.2
22
23
24
25
26
29
Allele repeat length

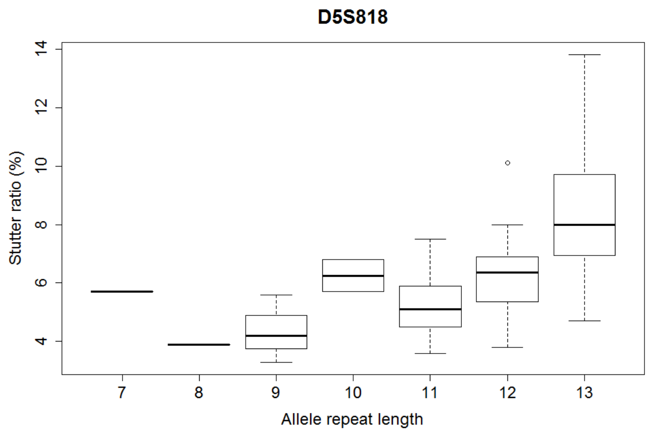
D5S818
Stutter ratio (%)
4 6 8 10 12 14
7 8 9 10 11 12 13
Allele repeat length

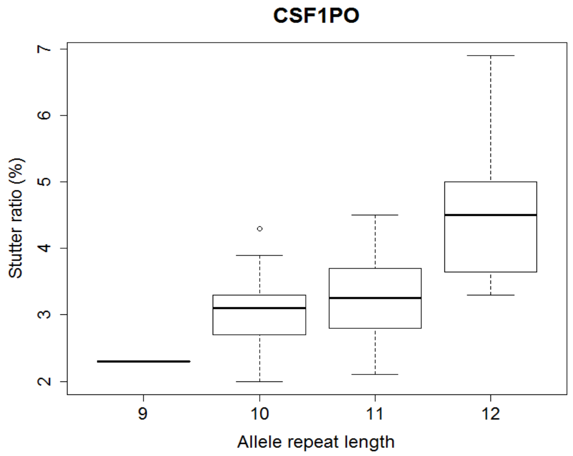
CSF1PO
Stutter ratio (%)
2 3 4 5 6 7
9 10 11 12
Allele repeat length

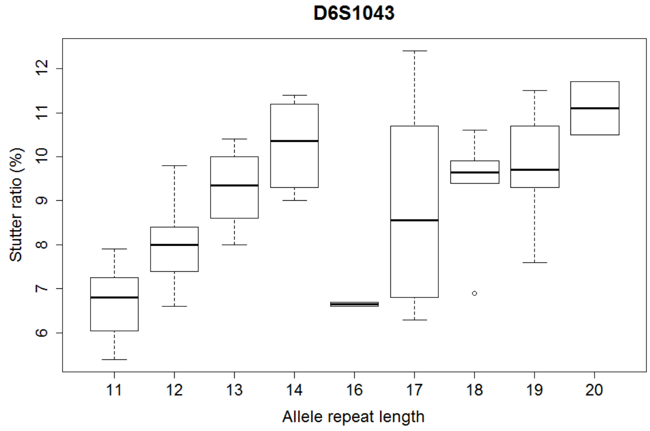
D6S1043
Stutter ratio (%)
6 7 8 9 10 11 12
11 12 13 14 16 17 18 19 20
Allele repeat length

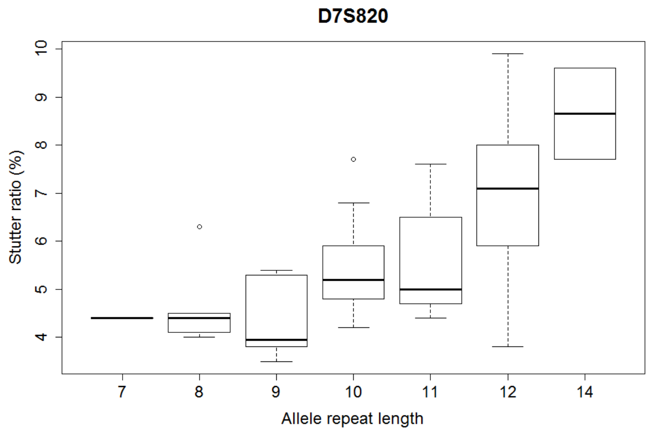
D7S820
Stutter ratio (%)
4 5 6 7 8 9 10
7 8 9 10 11 12 14
Allele repeat length

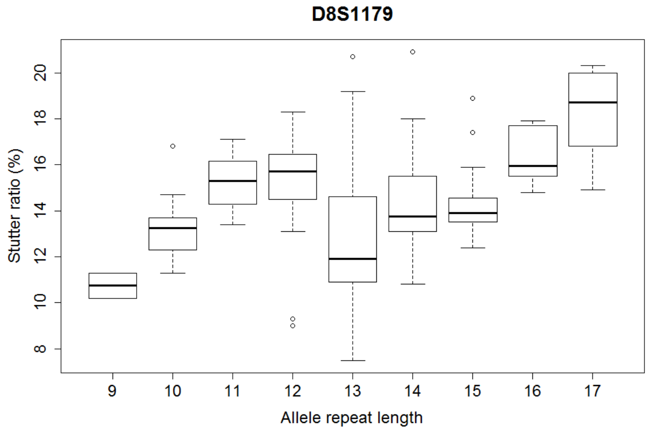
D8S1179
Stutter ratio (%)
8
10
12
14
16
18
20
9
10
11
12
13
14
15
16
17
Allele repeat length

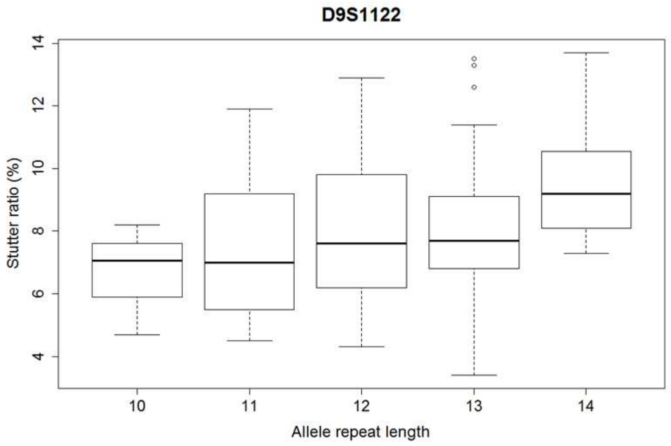
D9S1122
Stutter ratio (%)
4
6
8
10
12
14
10
11
12
13
14
Allele repeat length

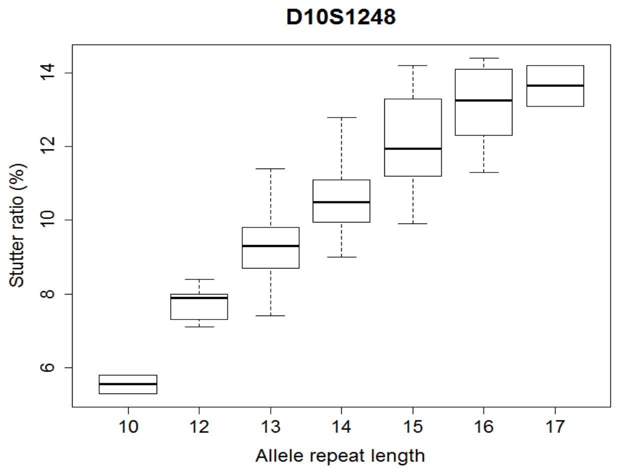
D10S1248
Stutter ratio (%)
6
8
10
12
14
10
12
13
14
15
16
17
Allele repeat length

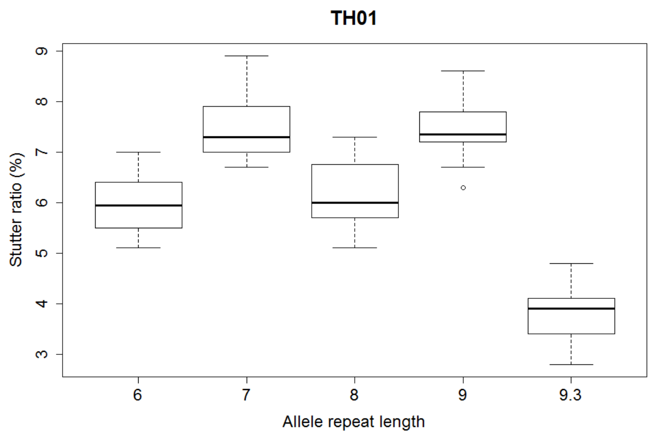
TH01
Stutter ratio (%)
3
4
5
6
7
8
9
6
7
8
9
9.3
Allele repeat length

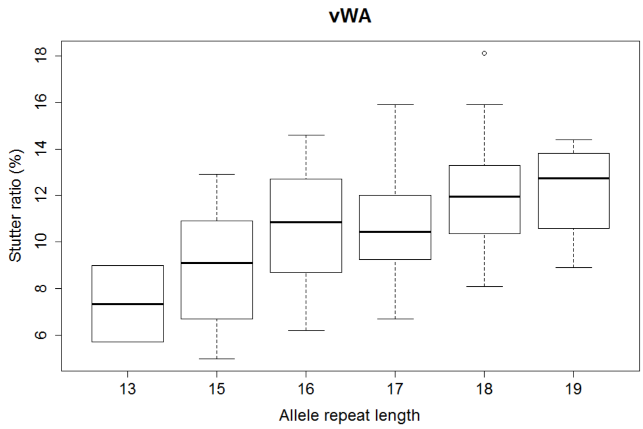
vWA
Stutter ratio (%)
6
8
10
12
14
16
18
13
15
16
17
18
19
Allele repeat length

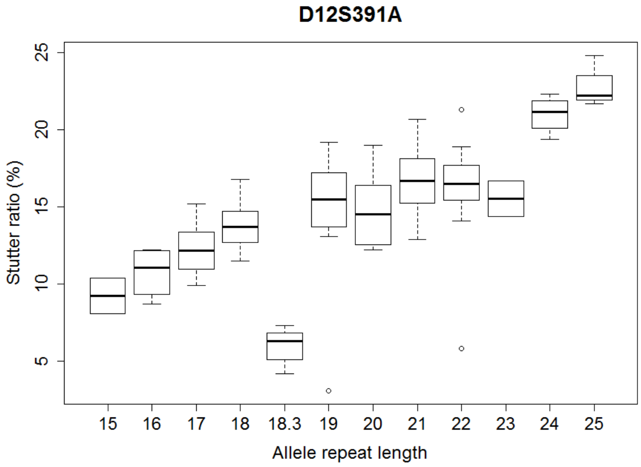
D12S391A
Stutter ratio (%)
5
10
15
20
25
15
16
17
18
18.3
19
20
21
22
23
24
25
Allele repeat length

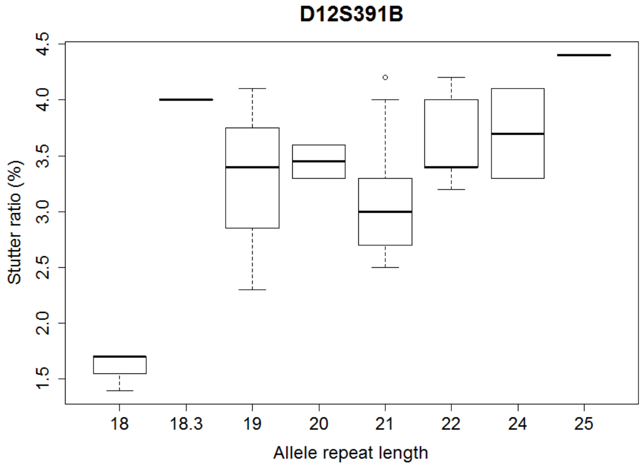
D12S391B
Stutter ratio (%)
1.5
2.0
2.5
3.0
3.5
4.0
4.5
18
18.3
19
20
21
22
24
25
Allele repeat length

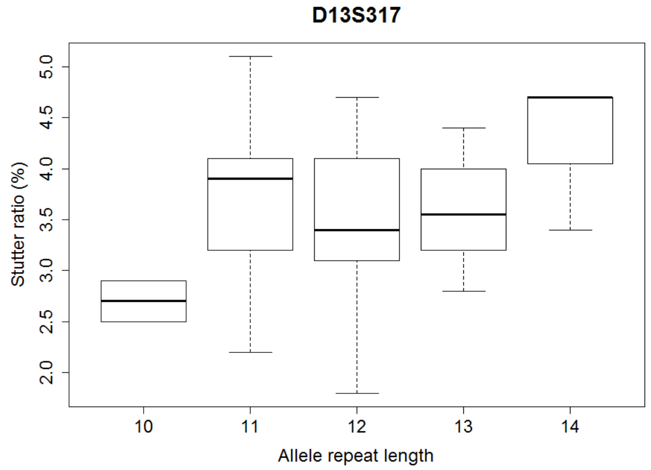
D13S317
Stutter ratio (%)
2.0
2.5
3.0
3.5
4.0
4.5
5.0
10
11
12
13
14
Allele repeat length

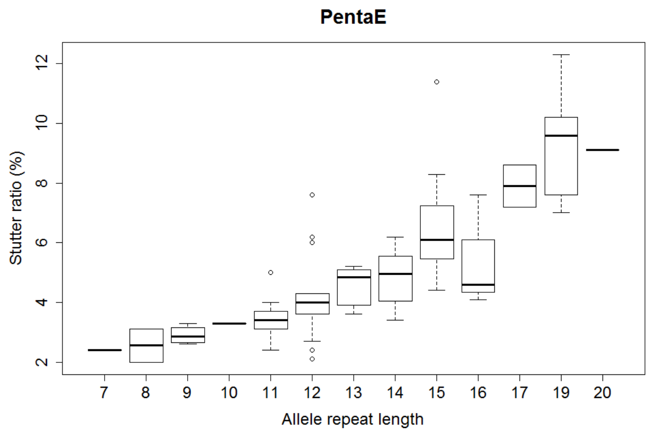
PentaE
Stutter ratio (%)
2
4
6
8
10
12
7
8
9
10
11
12
13
14
15
16
17
19
20
Allele repeat length

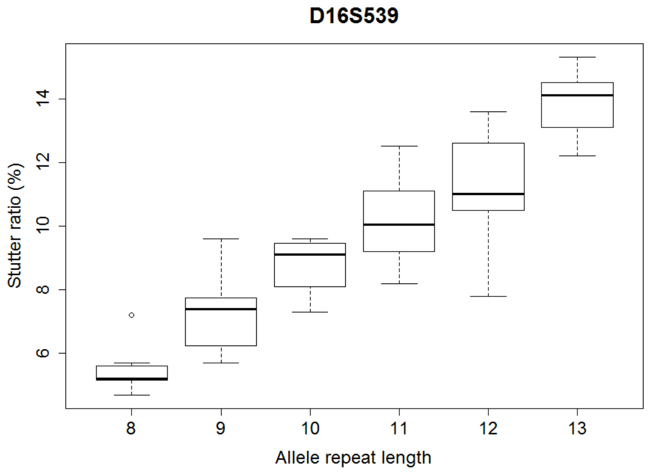
D16S539
Stutter ratio (%)
6
8
10
12
14
8
9
10
11
12
13
Allele repeat length

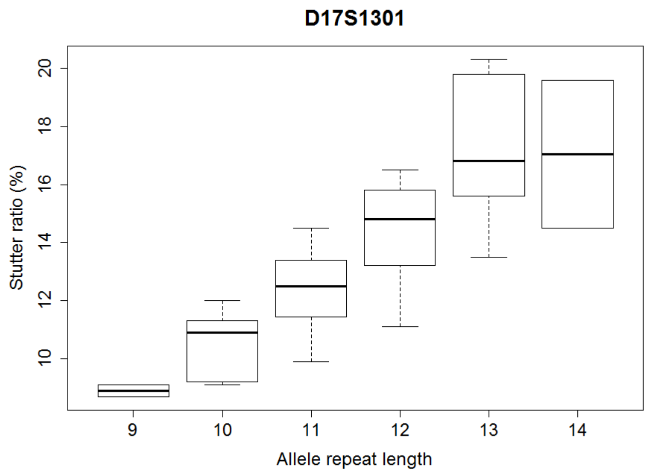
D17S1301
Stutter ratio (%)
10
12
14
16
18
20
9
10
11
12
13
14
Allele repeat length

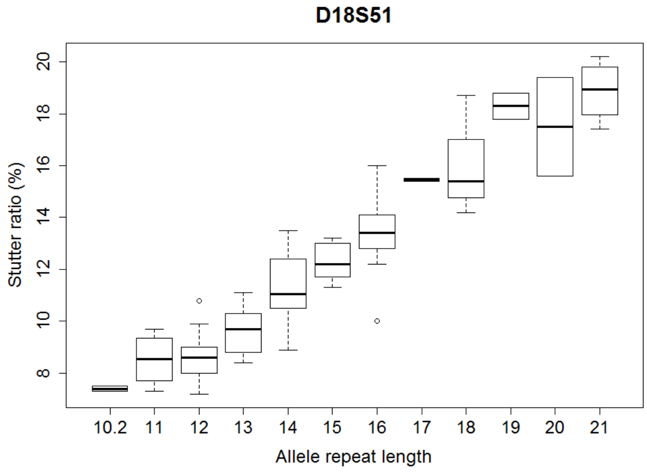
D18S51
Stutter ratio (%)
8
10
12
14
16
18
20
10.2
11
12
13
14
15
16
17
18
19
20
21
Allele repeat length

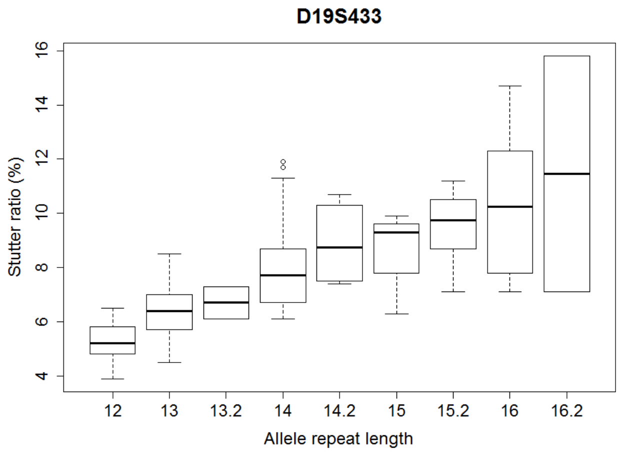
D19S433
Stutter ratio (%)
4
6
8
10
12
14
16
12
13
13.2
14
14.2
15
15.2
16
16.2
Allele repeat length

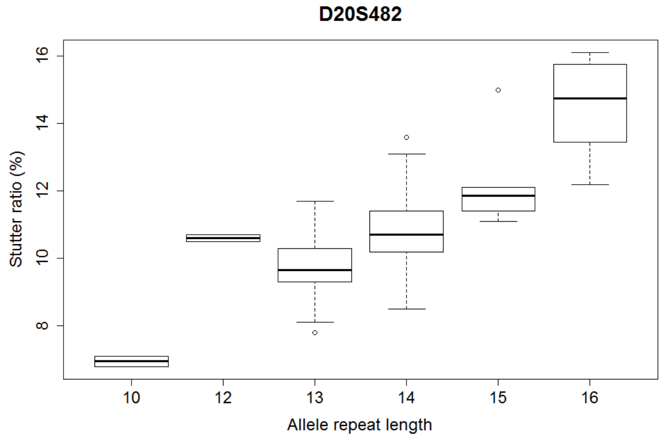
D20S482
Stutter ratio (%)
8
10
12
14
16
10
12
13
14
15
16
Allele repeat length

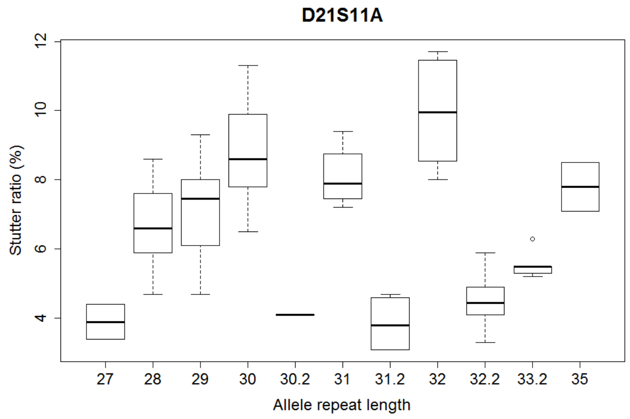
D21S11A
Stutter ratio (%)
4
6
8
10
12
27
28
29
30
30.2
31
31.2
32
32.2
33.2
35
Allele repeat length

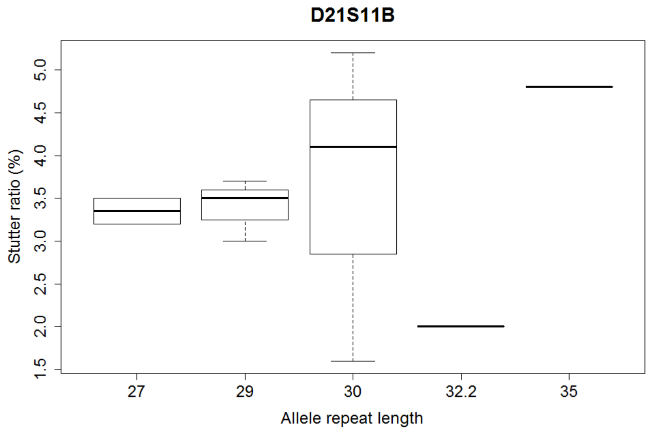
D21S11B
Stutter ratio (%)
1.5
2.0
2.5
3.0
3.5
4.0
4.5
5.0
27
29
30
32.2
35
Allele repeat length

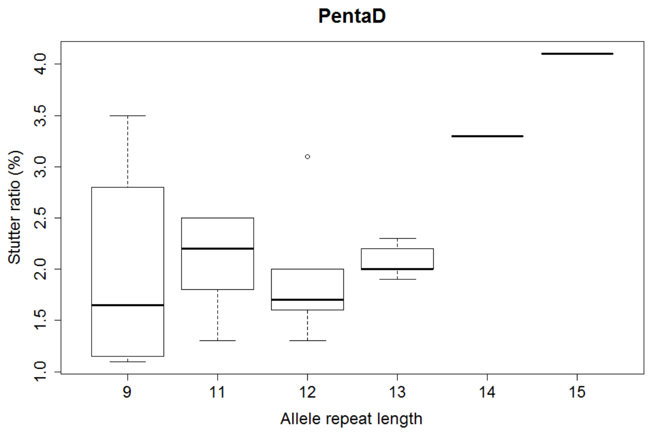
PentaD
Stutter ratio (%)
1.0
1.5
2.0
2.5
3.0
3.5
4.0
9
11
12
13
14
15
Allele repeat length

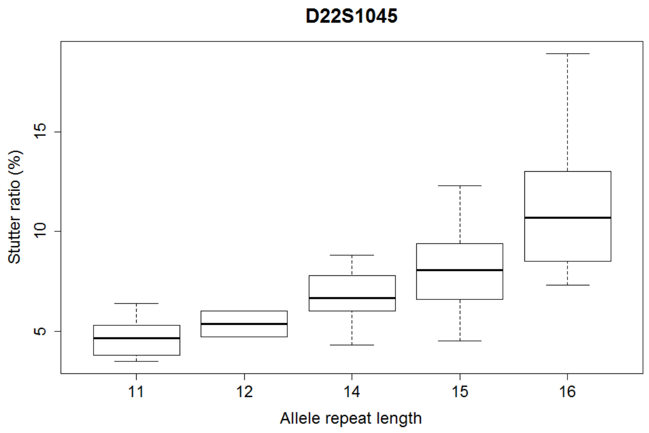
D22S1045
Stutter ratio (%)
5
10
15
11
12
14
15
16
Allele repeat length

X-STRs [a,b,c]

DXS10074

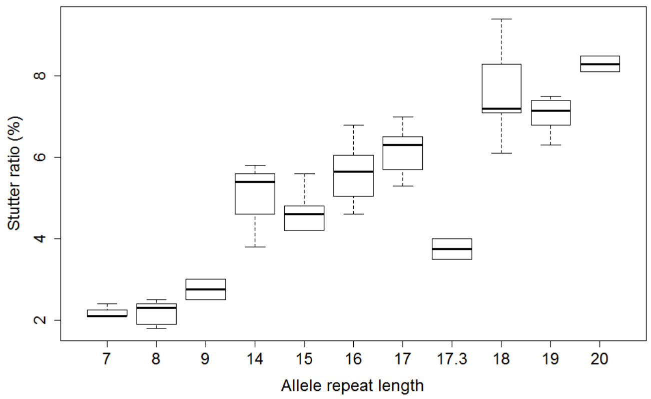

DXS10103

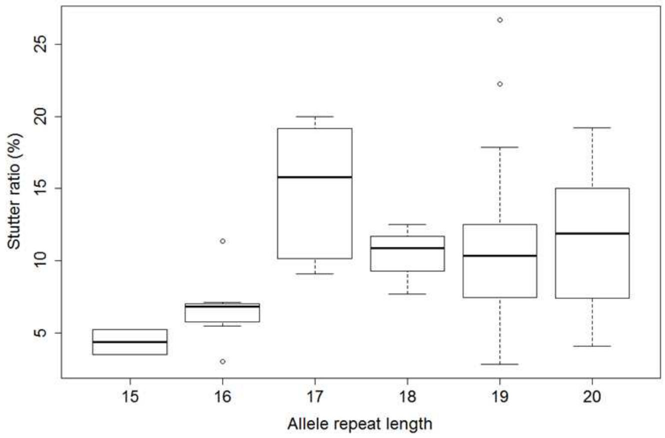

DXS10135

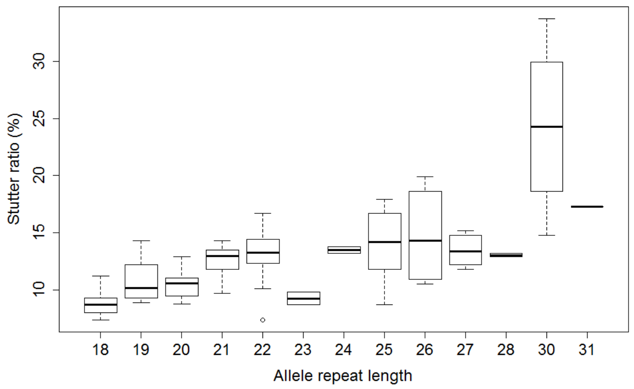

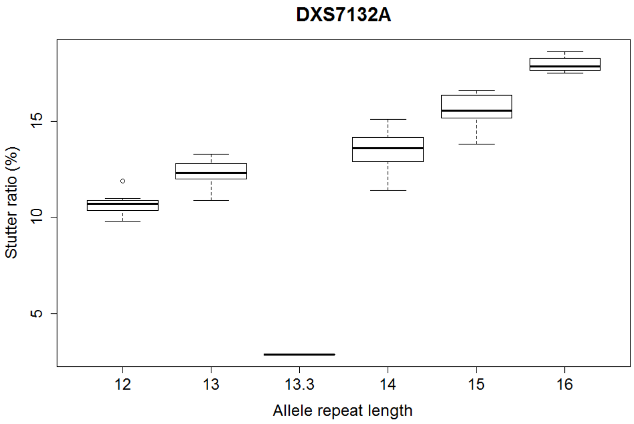
DXS7132A
Stutter ratio (%)
5
10
15
12
13
13.3
14
15
16
Allele repeat length

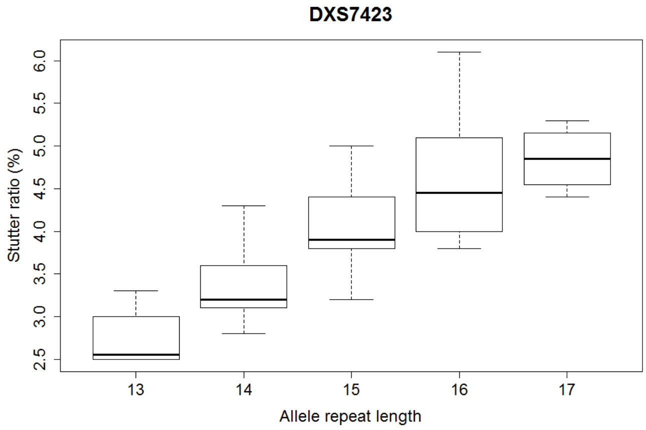
DXS7423
Stutter ratio (%)
2.5
3.0
3.5
4.0
4.5
5.0
5.5
6.0
13
14
15
16
17
Allele repeat length

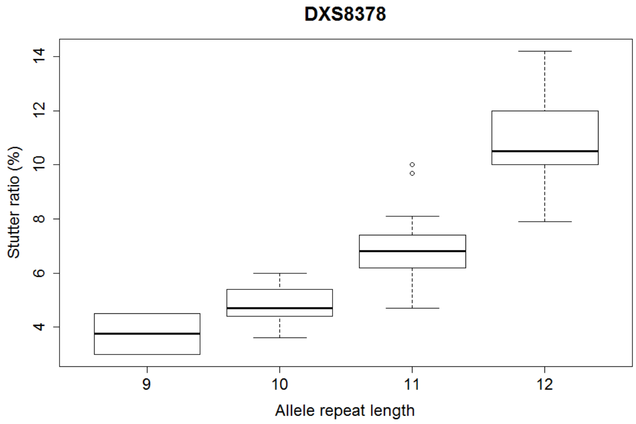
DXS8378
Stutter ratio (%)
4
6
8
10
12
14
9
10
11
12
Allele repeat length

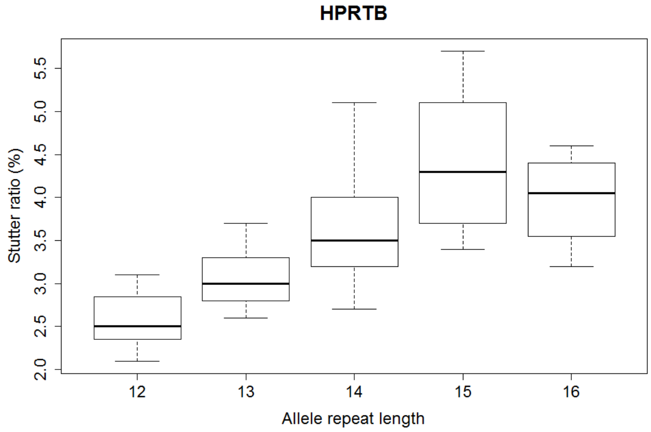
HPRTB
Stutter ratio (%)
2.0
2.5
3.0
3.5
4.0
4.5
5.0
5.5
12
13
14
15
16
Allele repeat length

Y-STRs [a,d]

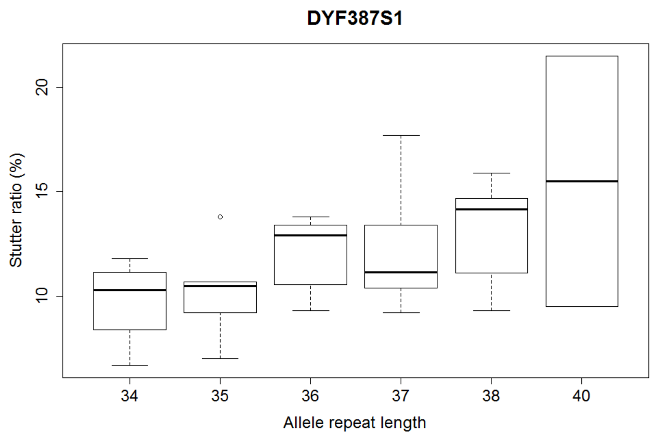
DYF387S1
Stutter ratio (%)
10
15
20
34
35
36
37
38
40
Allele repeat length

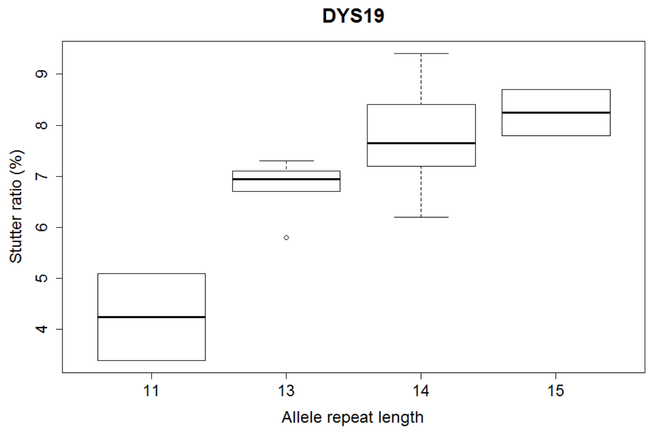
DYS19
Stutter ratio (%)
4
5
6
7
8
9
11
13
14
15
Allele repeat length

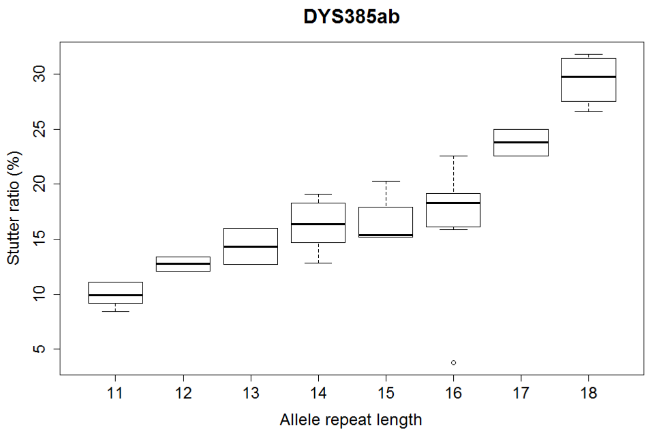
DYS385ab
Stutter ratio (%)
5
10
15
20
25
30
11
12
13
14
15
16
17
18
Allele repeat length

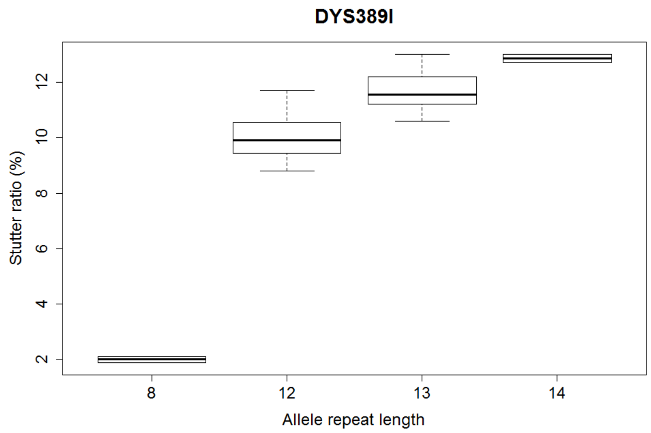
DYS389I
Stutter ratio (%)
2
4
6
8
10
12
8
12
13
14
Allele repeat length

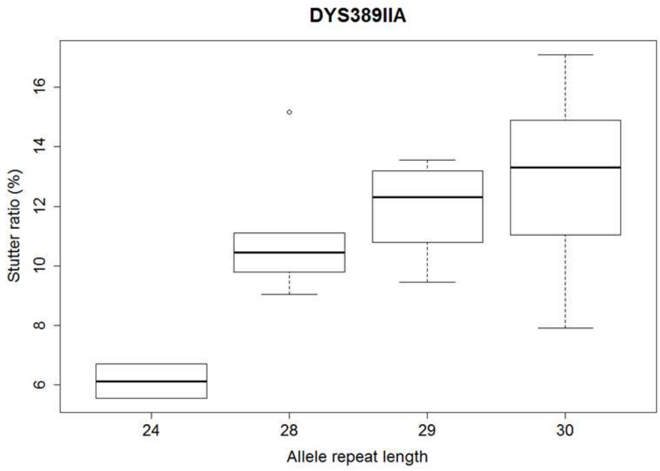
DYS389IIA
Stutter ratio (%)
6
8
10
12
14
16
24
28
29
30
Allele repeat length

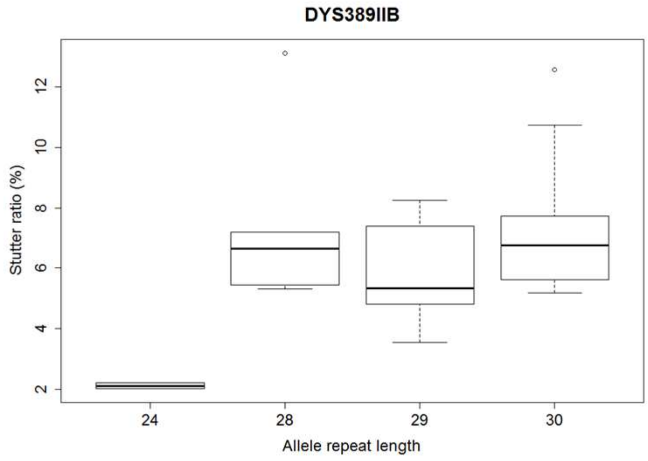
DYS389IIB
Stutter ratio (%)
2
4
6
8
10
12
24
28
29
30
Allele repeat length

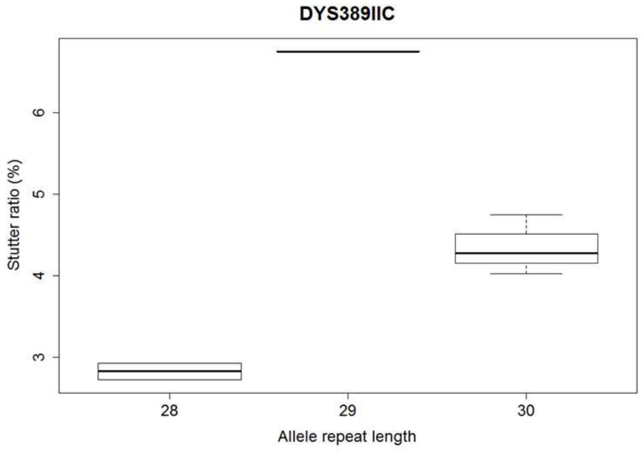
DYS389IIC
Stutter ratio (%)
3
4
5
6
28
29
30
Allele repeat length

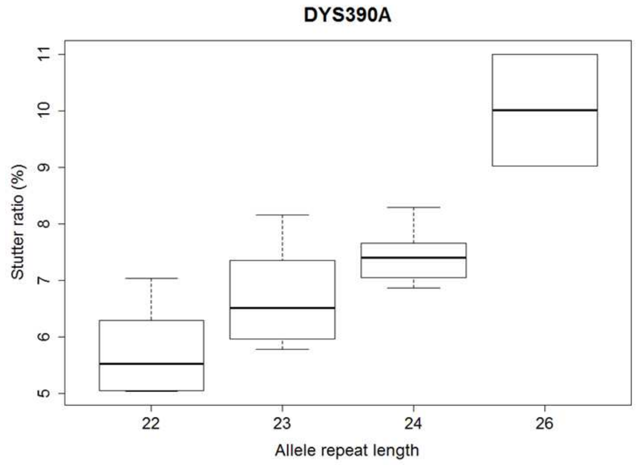
DYS390A
Stutter ratio (%)
5
6
7
8
9
10
11
22
23
24
26
Allele repeat length

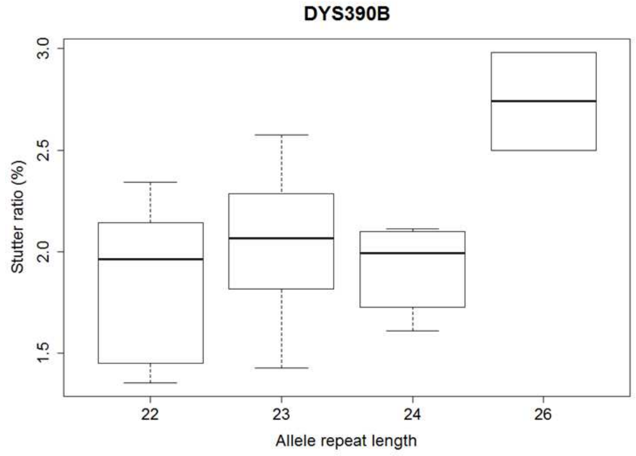
DYS390B
Stutter ratio (%)
1.5
2.0
2.5
3.0
22
23
24
26
Allele repeat length

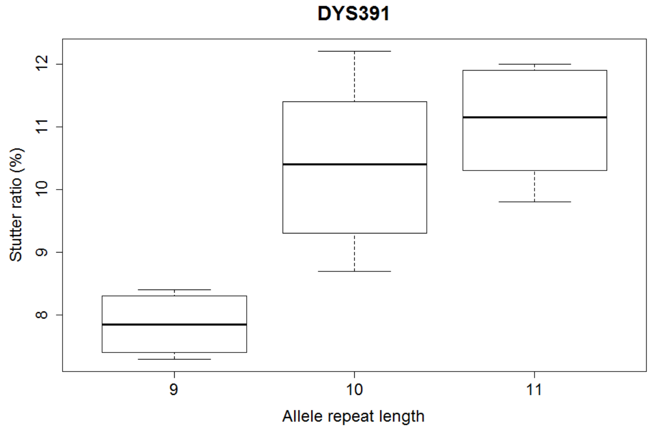
DYS391
Stutter ratio (%)
8
9
10
11
12
9
10
11
Allele repeat length

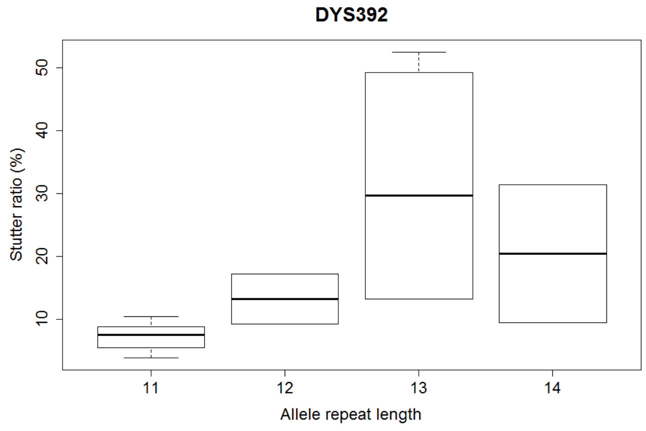
DYS392
Stutter ratio (%)
10
20
30
40
50
11
12
13
14
Allele repeat length

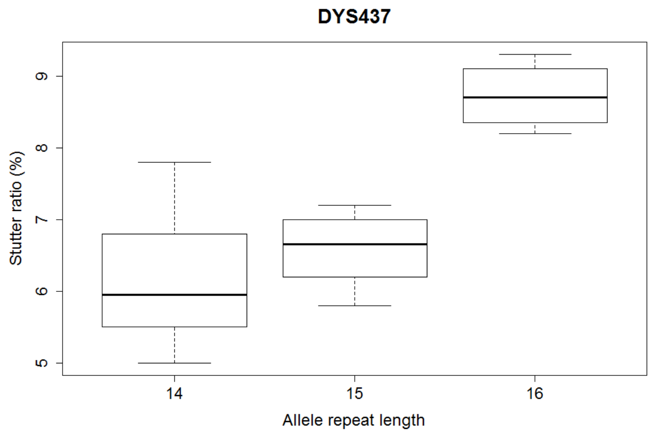
DYS437
Stutter ratio (%)
5
6
7
8
9
14
15
16
Allele repeat length

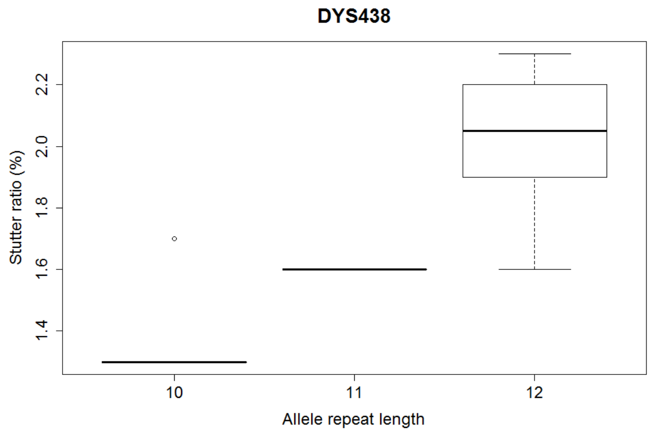
DYS438
Stutter ratio (%)
1.4
1.6
1.8
2.0
2.2
10
11
12
Allele repeat length

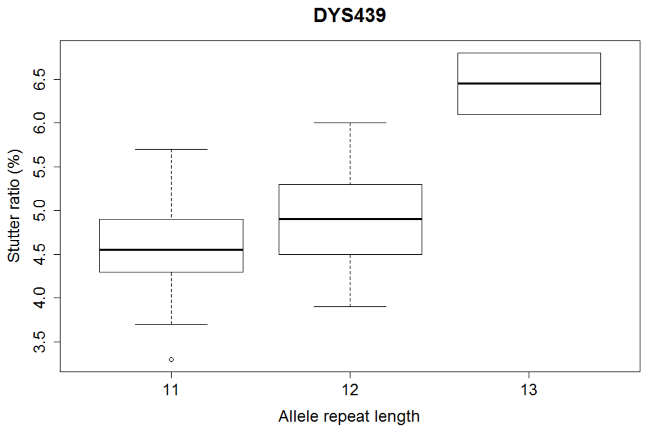
DYS439
Stutter ratio (%)
3.5
4.0
4.5
5.0
5.5
6.0
6.5
11
12
13
Allele repeat length

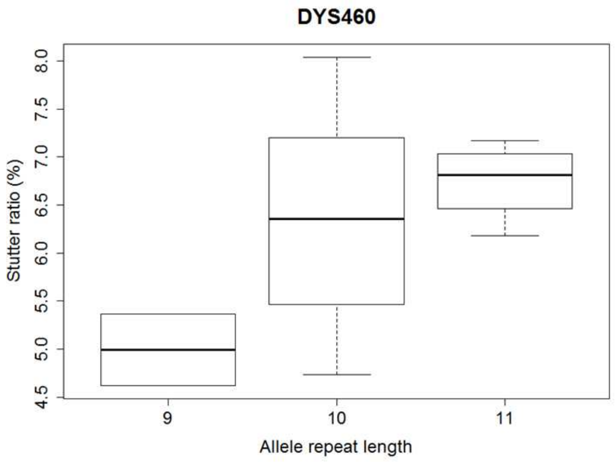
DYS460
Stutter ratio (%)
4.5
5.0
5.5
6.0
6.5
7.0
7.5
8.0
9
10
11
Allele repeat length

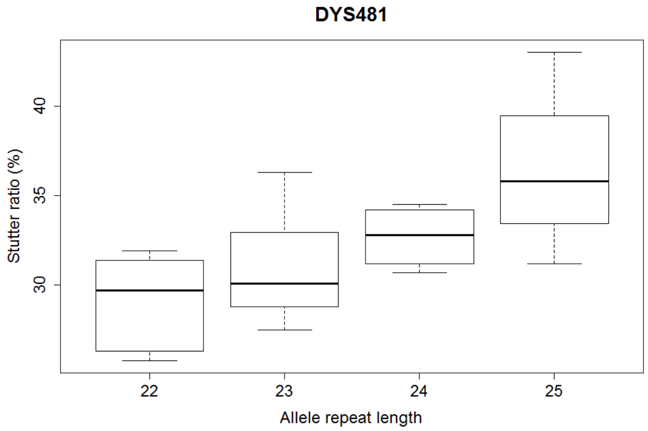
DYS481
Stutter ratio (%)
30
35
40
22
23
24
25
Allele repeat length

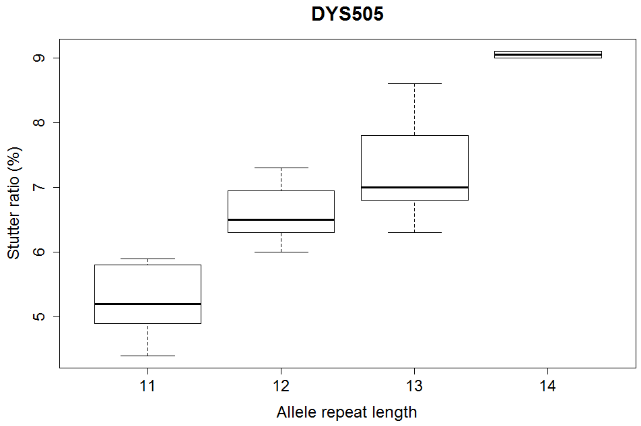
DYS505
Stutter ratio (%)
5
6
7
8
9
11
12
13
14
Allele repeat length

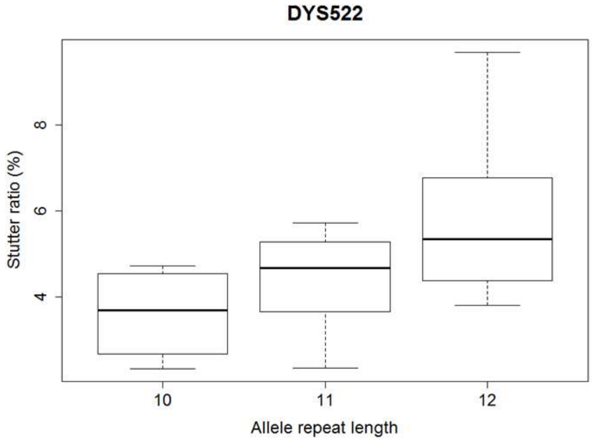
DYS522
Stutter ratio (%)
4
6
8
10
11
12
Allele repeat length

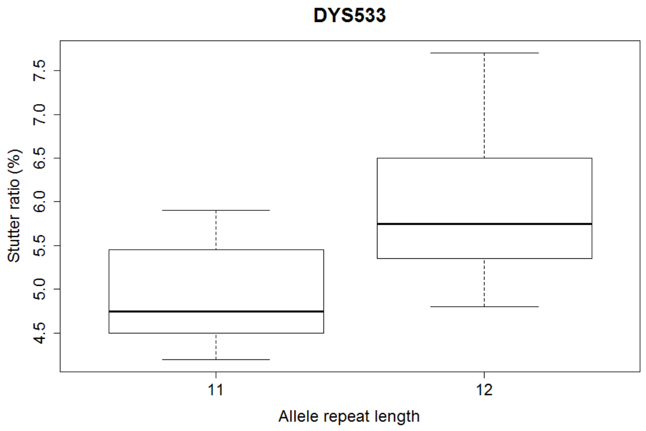
DYS533
Stutter ratio (%)
4.5
5.0
5.5
6.0
6.5
7.0
7.5
11
12
Allele repeat length

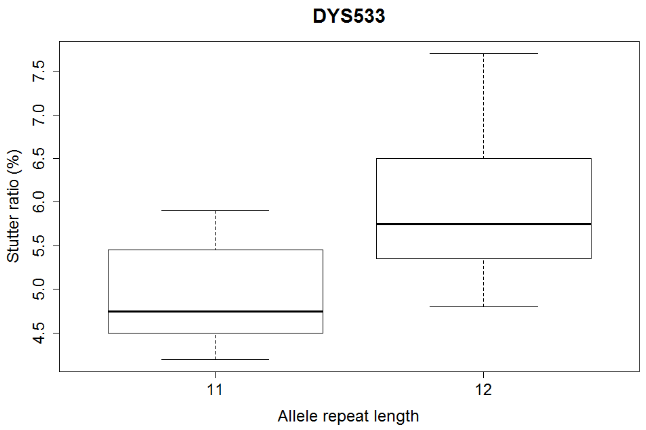
DYS533
Stutter ratio (%)
4.5
5.0
5.5
6.0
6.5
7.0
7.5
11
12
Allele repeat length

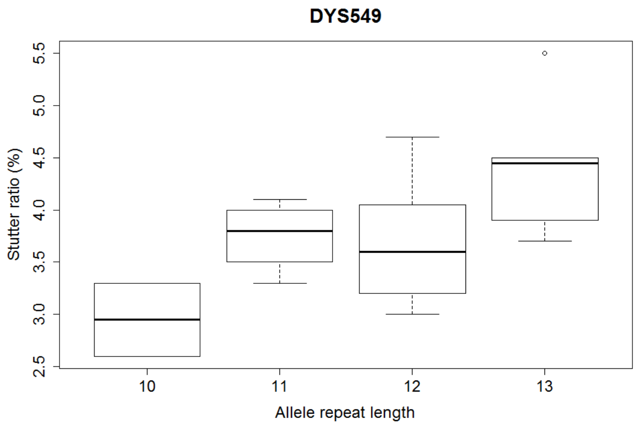
DYS549
Stutter ratio (%)
2.5
3.0
3.5
4.0
4.5
5.0
5.5
10
11
12
13
Allele repeat length

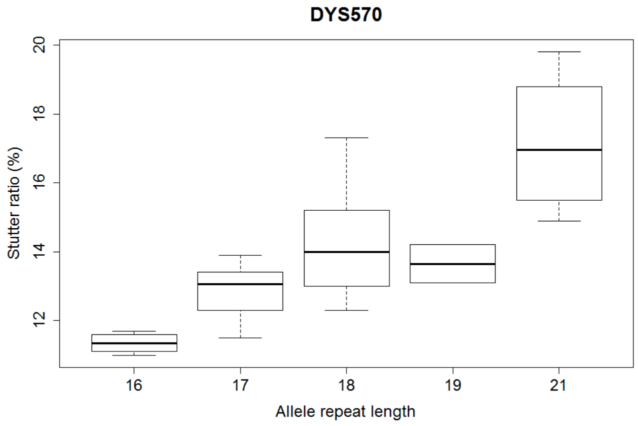
DYS570
Stutter ratio (%)
12
14
16
18
20
16
17
18
19
21
Allele repeat length

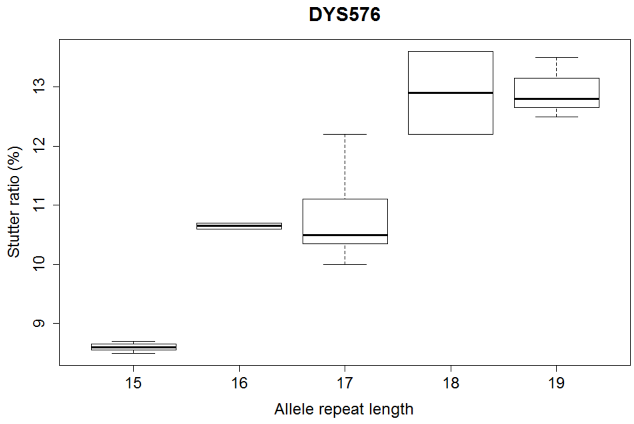
DYS576
Stutter ratio (%)
9
10
11
12
13
15
16
17
18
19
Allele repeat length

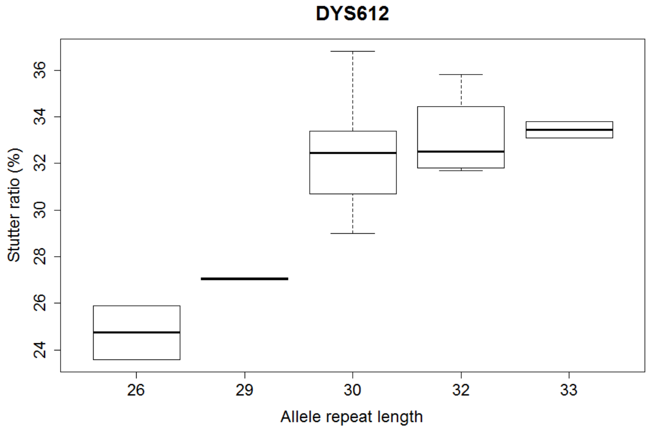
DYS612
Stutter ratio (%)
24
26
28
30
32
34
36
26
29
30
32
33
Allele repeat length

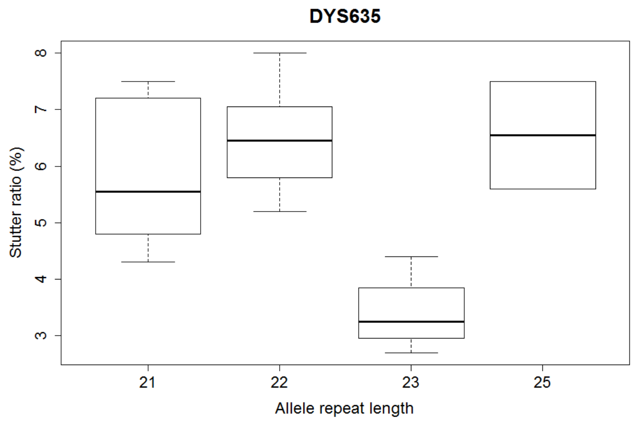
DYS635
Stutter ratio (%)
3
4
5
6
7
8
21
22
23
25
Allele repeat length

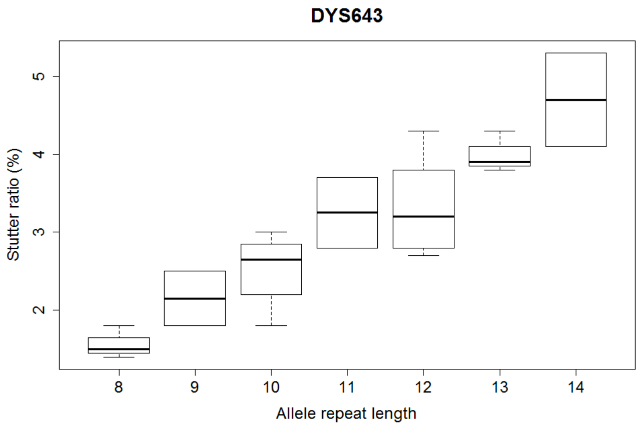
DYS643
Stutter ratio (%)
2
3
4
5
8
9
10
11
12
13
14
Allele repeat length

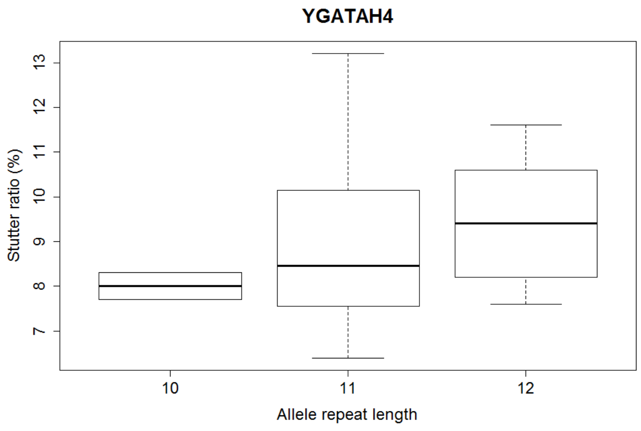
YGATAH4
Stutter ratio (%)
7
8
9
10
11
12
13
10
11
12
Allele repeat length

[a]When two different stutters were observed from the same mother allele, the one with the most reads was designated A, and the one with the least reads was designated B.

[b]A 3 bp deletion was detected in four alleles in the DXS10135 downstream flank. Their conventional CE allele designations were 17.1, 19.1, 20.1, 21.1, respectively. In order to find association between repeat length and stutter ratio, allele repeat lengths (not CE allele names) were used in the figure; thus, the alleles were designated 18, 20, 21, and 22, respectively.

[c]DXS7132 is composed of TAGA[+]. A microvariant allele DXS7132[13.3]TAGA[6]TGA[1]TAGA[7] gave the two stutters DXS7132[12.3]TAGA[5]tgaTAGA[7] (stutter A) and DXS7132[12.3]TAGA[6]tgaTAGA[6] (stutter B). The DXS7132[13.3] allele was the only microvariant observed.

[d]Stutter fractions of DYS461 were not assessed.
